# Inhibition of the Akt1-mTORC1 Axis Alters Venous Remodeling to Improve Arteriovenous Fistula Patency

**DOI:** 10.1038/s41598-019-47542-5

**Published:** 2019-07-30

**Authors:** Xiangjiang Guo, Arash Fereydooni, Toshihiko Isaji, Jolanta Gorecka, Shirley Liu, Haidi Hu, Shun Ono, Michelle Alozie, Shin Rong Lee, Ryosuke Taniguchi, Bogdan Yatsula, Naiem Nassiri, Lan Zhang, Alan Dardik

**Affiliations:** 1 Vascular Biology and Therapeutics Program, Yale School of Medicine, New Haven, CT USA; 2 Department of Vascular Surgery, Renji Hospital, School of Medicine, Shanghai Jiao Tong University, Shanghai, China; 3 Division of Vascular and Endovascular Surgery, Department of Surgery, Yale School of Medicine, New Haven, CT USA

**Keywords:** Restenosis, Mechanisms of disease

## Abstract

Arteriovenous fistulae (AVF) are the most common access created for hemodialysis, but up to 60% do not sustain dialysis within a year, suggesting a need to improve AVF maturation and patency. In a mouse AVF model, Akt1 regulates fistula wall thickness and diameter. We hypothesized that inhibition of the Akt1-mTORC1 axis alters venous remodeling to improve AVF patency. Daily intraperitoneal injections of rapamycin reduced AVF wall thickness with no change in diameter. Rapamycin decreased smooth muscle cell (SMC) and macrophage proliferation; rapamycin also reduced both M1 and M2 type macrophages. AVF in mice treated with rapamycin had reduced Akt1 and mTORC1 but not mTORC2 phosphorylation. Depletion of macrophages with clodronate-containing liposomes was also associated with reduced AVF wall thickness and both M1- and M2-type macrophages; however, AVF patency was reduced. Rapamycin was associated with improved long-term patency, enhanced early AVF remodeling and sustained reduction of SMC proliferation. These results suggest that rapamycin improves AVF patency by reducing early inflammation and wall thickening while attenuating the Akt1-mTORC1 signaling pathway in SMC and macrophages. Macrophages are associated with AVF wall thickening and M2-type macrophages may play a mechanistic role in AVF maturation. Rapamycin is a potential translational strategy to improve AVF patency.

## Introduction

With over half a million people affected by end-stage renal disease (ESRD) in the United States, the incidence of ESRD requiring hemodialysis is over 120,000 new cases per year^1^. Arteriovenous fistulae (AVF) remain the preferred conduit for vascular access, since AVF are associated with higher patency, lower risk of infection and greater long-term survival compared to arteriovenous grafts and central venous catheters^2–4^. Prior to successful use for hemodialysis, AVF must mature, that is thicken, dilate and increase flow, to support the high flow volumes and rates required for the dialysis session; however, AVF can fail to mature (“early” failure) in approximately 30–50% of patients^5^, preventing successful use of a patent conduit. Even if AVF do mature correctly, “late” failure occurs in 35–40% of patients with a functional AVF in the first year alone due to development of juxtaanastomotic neointimal hyperplasia^5,6^. These poor clinical outcomes reflect our imperfect understanding of mechanisms regulating successful venous remodeling in response to the fistula environment^6–8^.

We have previously shown that Akt1 expression is upregulated during venous remodeling, both during vein graft adaptation^9^, as well as during AVF maturation, a consistent response to two different hemodynamic environments^10^; during AVF maturation, Akt1 regulates both venous wall thickening as well as dilation, and is a mechanism of Eph-B4-mediated adaptive remodeling^10^. Mammalian target of rapamycin (mTOR) is a key regulatory protein that integrates signals from several pathways including the Akt1 pathway to modulate inflammation and coordinate cell growth and proliferation, all of which occur during venous remodeling^11^. Rapamycin, an mTOR inhibitor, is currently used for human therapy to prevent neointimal hyperplasia by reducing proliferation and migration of smooth muscle cells^12,13^. Since rapamycin inhibits Akt1 signaling^11^, and Akt1 mediates venous remodeling, we hypothesized that inhibition of the Akt1-mTORC1 axis with rapamycin reduces pathologic venous remodeling that is associated with AVF failure.

## Results

### Reduced AVF wall thickness, extracellular matrix deposition, SMC and macrophages with rapamycin

**Figure 1:**
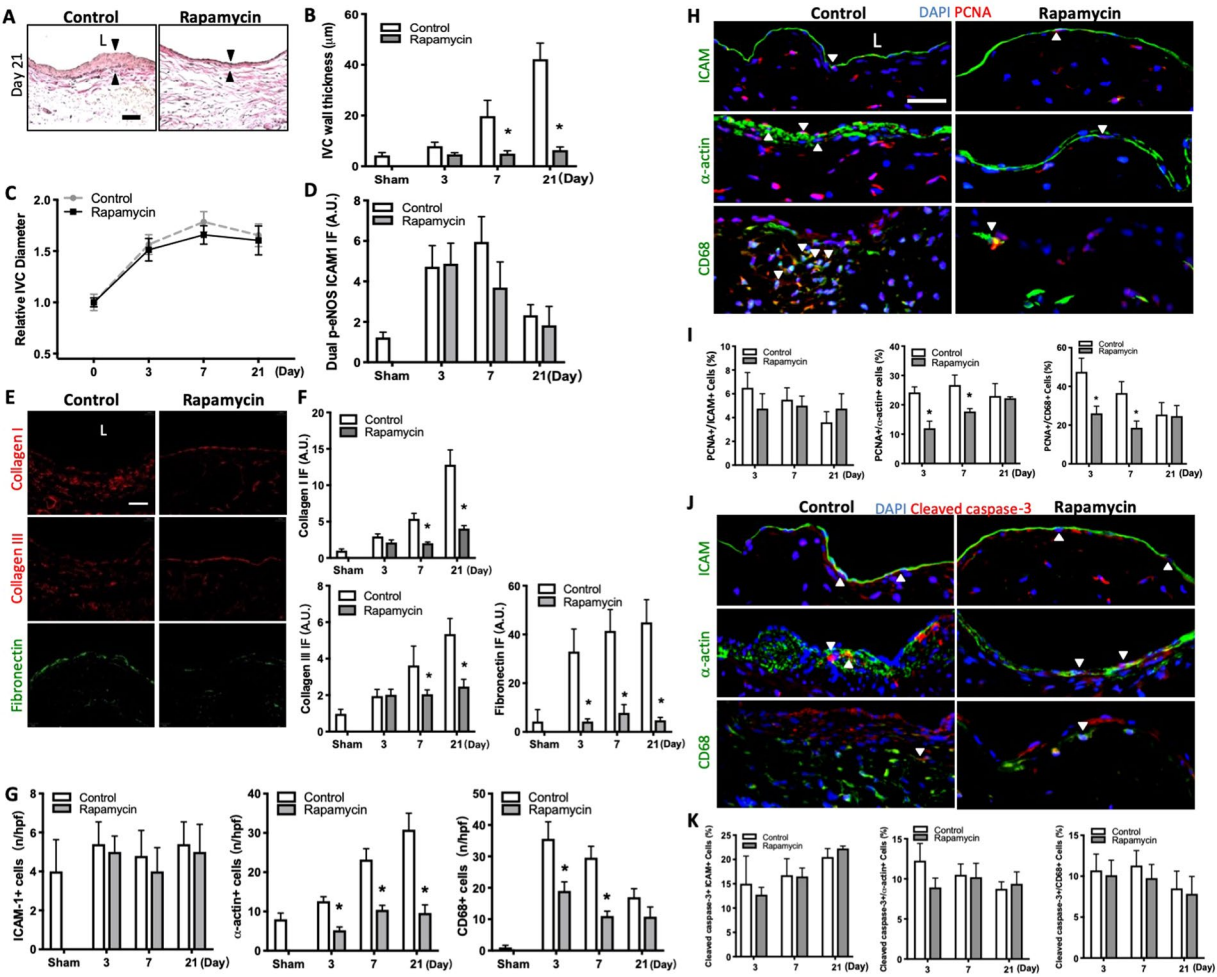
Reduced AVF wall thickness, extracellular matrix deposition, SMC and macrophages with rapamycin. (**A**) Representative photomicrographs showing AVF wall thickness in mice treated with rapamycin vs. control (day 21). Scale bar, 25 µm. L, lumen. (**B**) Bar graph showing AVF wall thickness in mice treated with rapamycin vs. control; p < 0.0001 (ANOVA); *p < 0.0001 (Sidak’s post hoc); n = 5–9. (**C**) Line graph showing relative AVF diameter in mice treated with rapamycin vs. control; normalized to day 0; p = 0.534 (ANOVA); n = 6. (**D**) Bar graphs showing quantification of dual IF after control or rapamycin treatment at days 3, 7, 21, normalized to sham. p-eNOS-ICAM1: p < 0.1383 (ANOVA); n = 4–6. (**E**) Photomicrographs showing representative of extracellular matrix immunoreactive signals in control or rapamycin treated groups (day 7). Collagen I or III (red) and fibronectin (green); scale bar, 25 μm. (**F**) Bar graphs showing quantification of IF, normalized to sham. Collagen I: p < 0.0001 (ANOVA); *p = 0.0006, day 7; *p < 0.0001, day 21 (post hoc); n = 4. Collagen III: p < 0.0001 (ANOVA); *p = 0.0122, day 7; *p < 0.0001, day 21 (post hoc); n = 4. Fibronectin: p < 0.0001 (ANOVA); *p < 0.0001 (post hoc); n = 5. (**G**) Bar graphs showing number of ICAM-1^+^, α-actin^+^ or CD68^+^ cells in AVF after control or rapamycin treatment. ICAM-1: p = 0.7455 (ANOVA). n = 5. α-actin: p < 0.0001 (ANOVA). *p < 0.0002, day 3; *p < 0.0001, day 7; *p < 0.0001, day 21 (post hoc); n = 5. CD68: p < 0.0001 (ANOVA). *p < 0.0001, days 3 and 7; *p = 0.0463, day 21 (post hoc); n = 5. (**H**) Photomicrographs showing representative IF of PCNA (red) merged with ICAM, α-actin or CD68 (green), and DAPI (blue) in AVF of control vs rapamycin treated mice (day 7); L, lumen; scale bar, 25 μm. White arrowheads indicate merged signal. (**I**) Bar graphs showing percentage of dual positive cells. PCNA-ICAM: p = 0.4137 (ANOVA). n = 4–5. PCNA-α-actin: p < 0.0001 (ANOVA). *p < 0.0001, day 3; *p = 0.0011, day 7 (post hoc); n = 4–5. PCNA-CD68: p < 0.0001 (ANOVA). *p = 0.0002, day 3; *p = 0.0023, day 7 (post hoc); n = 4–5. (**J**) Photomicrographs showing representative IF of cleaved caspase-3 (red) merged with ICAM, α-actin or CD68 (green), and DAPI (blue) in AVF of control or rapamycin treated mice (day 7); L, lumen; scale bar, 25 μm. White arrowheads indicate merged signal. (**K**) Bar graphs showing percentage of dual positive cells. Cleaved caspase-3-ICAM: p = 0.08777 (ANOVA); n = 4–5. Middle graph, cleaved caspase-3-α-actin: p = 0.1266 (ANOVA). n = 4–5. Right graph, cleaved caspase-3-CD68: p = 0.2663 (ANOVA); n = 4–5.

To determine the effects of mTOR signaling during venous remodeling such as occurs during AVF maturation, we used a mouse model of AVF that recapitulates human AVF maturation^14^. Aortocaval fistulae were created as previously described and afterwards mice received daily intraperitoneal (IP) injections of rapamycin (100 μg) or vehicle alone; in mice treated with rapamycin, rapamycin was detectable in serum without any systemic signs of immunosuppression or toxicity (Supplemental Fig. 1A). The IVC of sham-operated and fistula of control-treated and rapamycin-treated mice were harvested and analyzed on postoperative days 3, 7 and 21 (Supplemental Fig. 1B). Compared to sham-operated mice, control AVF showed wall thickening that was reduced in AVF treated with rapamycin (Fig. 1A,B; Supplemental Fig. 1C,D); however, there was no significant difference in the dilation of the IVC (Fig. 1C) or the aorta (Supplemental Fig. 1E), as well as immunoreactivity of p-eNOS-ICAM dual-positive cells (Fig. 1D; Supplemental Fig. 1F), between rapamycin-treated and control groups. Since rapamycin treatment reduced AVF wall thickening, we determined the effect of rapamycin on components of the AVF wall including several extracellular matrix (ECM) proteins as well as endothelial cells (EC)^15,16^, smooth muscle cells (SMC)^10,17,18^, and macrophages^17,19,20^. There was reduced immunoreactivity of collagen I, collagen III, and fibronectin in the AVF wall of rapamycin-treated mice, compared to control mice (Fig. 1E,F; Supplemental Fig. 1G). There were fewer numbers of α-actin-positive cells and CD68-positive cells, without any change in numbers of intercellular adhesion molecule-1 (ICAM-1)-positive cells, in the AVF of rapamycin-treated mice compared to control mice, consistent with reduced numbers of SMC and macrophages but not EC with rapamycin treatment (Fig. 1G; Supplemental Fig. 1H). The reduced number of α-actin-positive cells and CD68-positive cells with rapamycin treatment was associated with reduced PCNA immunoreactivity (Fig. 1H,I; Supplemental Fig. 1I); however, there was no increase in cleaved caspase-3 immunoreactivity with rapamycin treatment (Fig. 1J,K; Supplemental Fig. 1J). These data suggest that the reduced AVF wall thickening with rapamycin treatment is associated with less SMC and macrophage proliferation.

### Reduced M1- and M2-type macrophages with rapamycin

**Figure 2:**
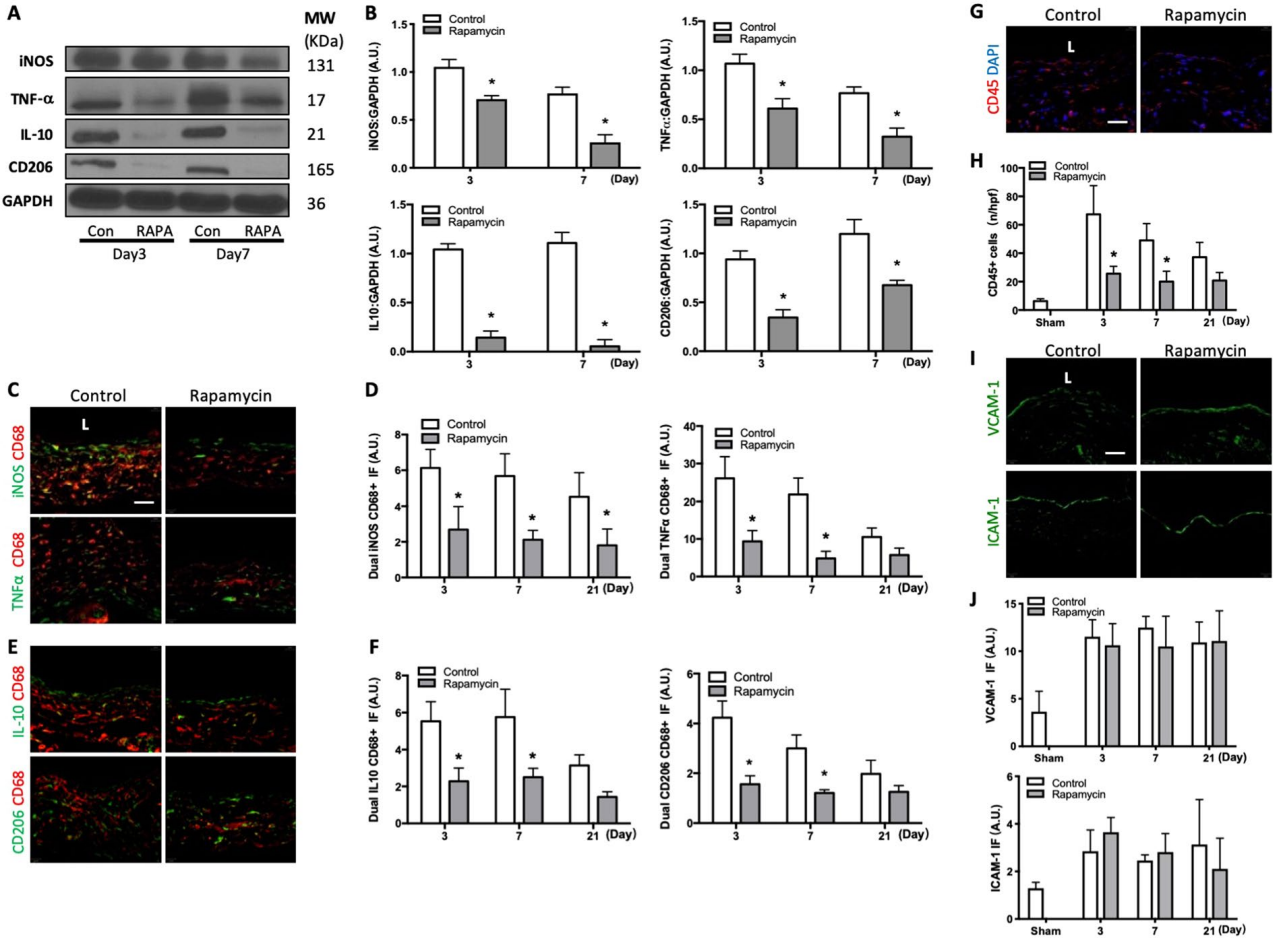
Reduced M1- and M2-type macrophages with rapamycin. (**A**) Representative Western blot showing iNOS, TNF-α, IL-10 and CD206 protein expression levels in AVF treated with rapamycin or control (day 3 and 7). (**B**) Graphs showing densitometry measurements of iNOS, TNF-α, IL-10 and CD206 expression in the AVF after control or rapamycin treatment, normalized to GAPDH. iNOS: p = 0.0011 (ANOVA). *p = 0.0241, day 3; *p = 0.0054, day 7 (post hoc); n = 2–3. TNF-α: *p = 0.0020 (ANOVA). *p = 0.0223, day 3; *p = 0.0250, day 7 (post hoc); n = 2–3. IL-10: *p < 0.0001 (ANOVA). *p = 0.0011, day 3; *p = 0.0006, day 7 (post hoc); n = 2–3. CD206: p = 0.0013 (ANOVA). *p = 0.0126, day 3; *p = 0.0200, day 7 (post hoc); n = 2–3. (**C**) Photomicrographs showing representative dual IF for CD68 (red) and iNOS (green, top row) or CD68 (red) and TNF-α (green, bottom row) in AVF after control or rapamycin treatment (day 7). Scale bar, 25 μm. L, lumen. (**D**) Bar graphs showing quantification of dual IF after control or rapamycin treatment. Left graph, iNOS-CD68: p < 0.0001 (ANOVA). *p = 0.0006, day 3; *p = 0.0004, day 7; *p = 0.0073, day 21 (post hoc); n = 5. Right graph, TNF-α-CD68: p < 0.0001 (ANOVA). *p < 0.0001, day 3; *p < 0.0001, day 7 (post hoc); n = 5. (**E**) Photomicrographs showing representative dual IF for CD68 (red) and IL-10 (green, top row) and CD68 (red) and CD206 (green, bottom row) in control or rapamycin treated AVF (day 7). (**F**) Bar graphs showing quantification of dual IF after control or rapamycin treatment (day 7). Left graph, IL-10-CD68: p < 0.0001 (ANOVA). *p < 0.0001, day 3; *p < 0.0001, day 7 (post hoc); n = 5. CD206-CD68: p < 0.0001 (ANOVA). *p < 0.0001, day 3; *p < 0.0001, day 7 (post hoc); n = 5. (**G**) Photomicrograph of representative of CD45+ cells in control or rapamycin treated mice AVF (day 7). (**H**) Bar graph showing number of CD45 immunoreactive cells in AVF after control vs rapamycin treatment; p < 0.0001 (ANOVA); *p < 0.0001, day 3; *p = 0.0020, day 7; *p = 0.2110, day 21 (post hoc); n = 5. (**I**) Representative photomicrographs showing VCAM-1 (top row) and ICAM-1 (bottom row) IF in AVF after control or rapamycin treatment (day 7). (**J**) Bar graphs showing relative quantification of VCAM-1 and ICAM-1 intensity in AVF, normalized to sham vessels. VCAM-1: p = 0.3162 (ANOVA); n = 6. ICAM-1: p = 0.9280 (ANOVA); n = 4–6. Data represent mean ± SEM.

Since rapamycin treatment was associated with reduced macrophage proliferation (Fig. 1), we determined whether rapamycin had differential effects on macrophage subpopulations. The wall of the rapamycin-treated AVF showed decreased iNOS and TNF-α immunoreactive protein, markers of M1-type macrophages, as well as decreased IL-10 and CD206 protein, markers of M2-type macrophages, at both days 3 and 7 (Fig. 2A,B). Rapamycin-treated AVF also showed reduced immunoreactivity of CD68-iNOS dual-positive cells as well as CD68-TNF-α dual-positive cells in the adventitia (Fig. 2C,D; Supplemental Fig. 2A); there was also reduced immunoreactivity of CD68-IL-10 dual-positive cells as well as CD68-CD206 dual-positive cells in the adventitia, at both days 3 and 7 (Fig. 2E,F; Supplemental Fig. 2B). Rapamycin treatment was also associated with fewer number of leukocyte common antigen (CD45) immunoreactive cells (Fig. 2G,H; Supplemental Fig. 2C), but there was no difference in immunoreactivity of vascular cell adhesion molecule-1 (VCAM-1) or ICAM-1 (Fig. 2I,J; Supplemental Fig. 2D). These data suggest that rapamycin is associated with reduced immunoreactivity of both M1-type and M2-type macrophages as well as fewer leukocytes during AVF remodeling.

### Reduced Akt1 and mTORC1 but not mTORC2 phosphorylation with rapamycin

**Figure 3:**
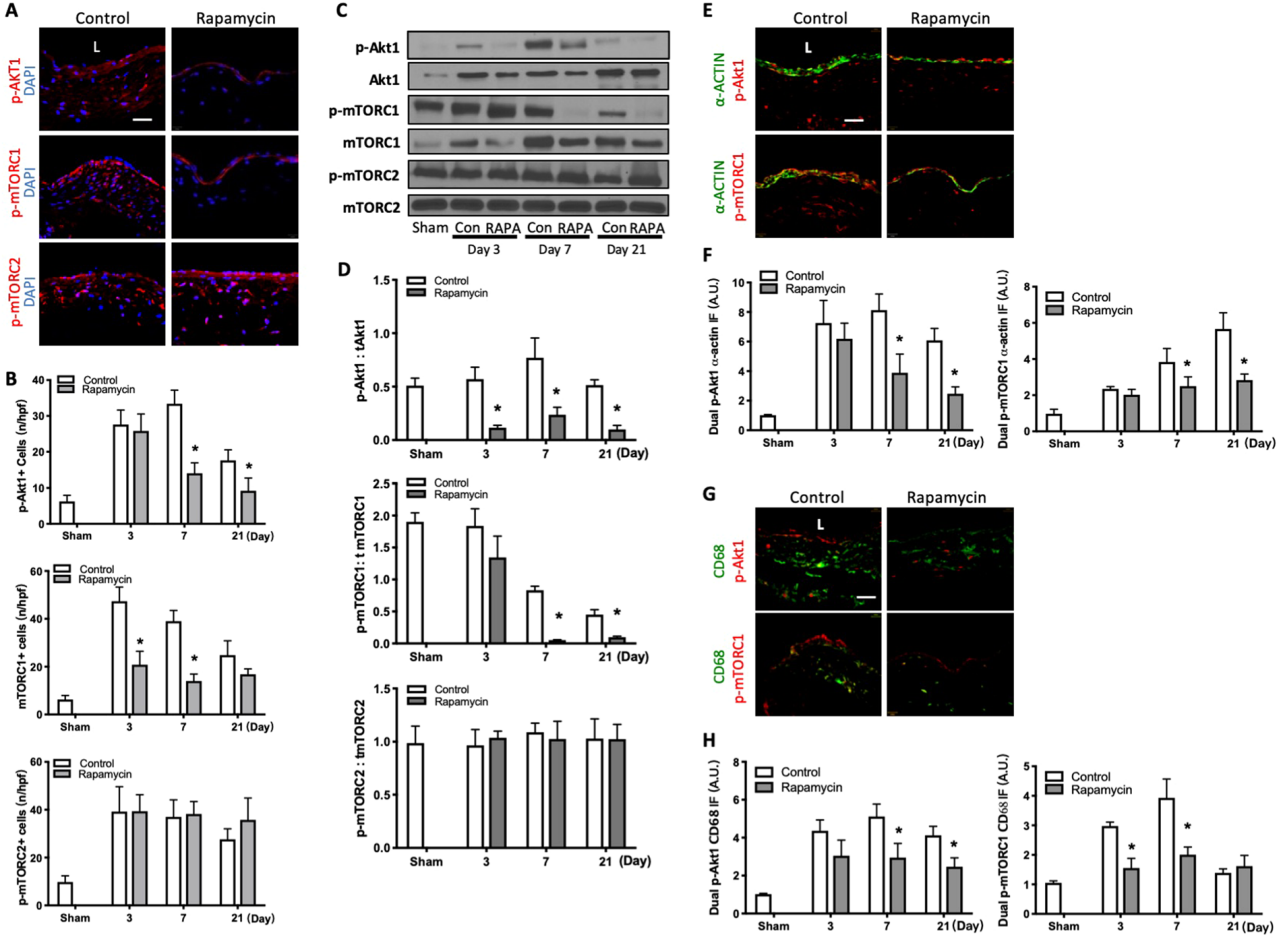
Reduced Akt1 and mTORC1 but not mTORC2 phosphorylation with rapamycin. (**A**) Photomicrographs showing representative IF of p-Akt1+ (top, red), p-mTORC1+ (middle, red) and p-mTORC2+ (bottom, red) cells in control or rapamycin treated mice AVF (day 7). Scale bar, 25μm. L, lumen. (**B**) Bar graphs showing number of p-Akt1+, p-mTORC1+ and p-mTORC2+ cells in AVF after rapamycin or control treatment. p-Akt-1: *p < 0.0001 (ANOVA); *p < 0.0001, day 7; *p = 0.0105, day 21 (post hoc); n = 4–5. p-mTORC1: p < 0.0001 (ANOVA); *p < 0.0001, day 3; *p < 0.0001, day 7 (post hoc); n = 4–5. p-mTORC2: p = 0.2870 (ANOVA); n = 4–5. (**C**) Representative Western blot showing Akt1, mTORC1, mTORC2 phosphorylation level after control vs rapamycin treatment. (**D**) Graphs showing densitometry measurement of Akt1, mTORC1 and mTORC2 phosphorylation. p-Akt1: tAkt1, p = 0.0002 (ANOVA); *p = 0.0110, day 7; *p = 0.0359, day 21 (post hoc); n = 3. p-mTORC1: tmTORC1, p = 0.0004 (ANOVA); *p = 0.0157, day 3; *p = 0.0192, day 7; *p = 0.0366, day 21 (post hoc); n = 3. p-mTORC2: tmTORC2: P = 0.9893 (ANOVA); n = 3. (**E**) Photomicrographs showing representative IF of dually-positive α-actin (green) and p-Akt1 (red, first row) or p-mTORC1 (red, second row) in AVF after control or rapamycin treatment (day 7). (**F**) Bar graphs showing quantification of dual IF after control vs rapamycin treatment. P-Akt1-α-actin: p < 0.0001 (ANOVA); *p = 0.0002, day 7; *p = 0.0017, day 21 (post hoc); n = 4–5. p-mTORC1-α-actin: p < 0.0001 (ANOVA); *p = 0.0136, day 7; *p < 0.0001, day 21 (post hoc); n = 4. (**G**) Photomicrographs showing representative dual IF for CD68 (green) and p-Akt1 (red, top row) or p-mTORC1 (red, bottom row) in AVF after control or rapamycin treatment (day 7). (**H**) Bar graphs showing quantification of dual IF after control vs rapamycin treatment. p-Akt1-CD68: p < 0.0001 (ANOVA); *p = 0.0013, day 7; *p = 0.0183, day 21 (post hoc); n = 4–5. p-mTORC1-CD68: p < 0.0001 (ANOVA); *p < 0.0001, day 3; *p < 0.0001, day 7 (post hoc); n = 4–5. Data represent mean ± SEM.

Since mTOR binds to either the Raptor regulatory subunit to form mTORC1, a downstream target of Akt1^21^, or to the Rictor regulatory subunit to form mTORC2^22^, an upstream regulator of Akt1^11^, we next determined whether rapamycin altered the phosphorylation of either of these complexes during AVF remodeling. Rapamycin was associated with reduced numbers of p-Akt1 immunoreactive cells (days 7 and 21) and p-mTORC1 immunoreactive cells (days 3 and 7), but there was no difference in the numbers of p-mTORC2 immunoreactive cells (Fig. 3A,B; Supplemental Fig. 3A). Similarly, mice treated with rapamycin had decreased expression of phosphorylated Akt1 and phosphorylated mTORC1, with no significant change in expression of phosphorylated mTORC2, in the AVF wall (days 3–21; Fig. 3C,D). Reduced Akt1 and mTORC1 phosphorylation with rapamycin was similarly reduced in both p-Akt1-α-actin dual-positive cells as well as p-mTORC1-α-actin dual-positive cells (Fig. 3E,F; Supplemental Fig. 3D); immunoreactivity was also reduced with rapamycin treatment in p-Akt1-CD68 dual-positive cells as well as p-mTORC1-CD68 dual-positive cells (Fig. 3G,H; Supplemental Fig. 3E). However, there was no significant difference in immunoreactivity of p-Akt1-ICAM dual-positive cells or p-mTORC1-ICAM dual-positive cells with rapamycin treatment (Supplemental Fig. 3B,C). These data suggest that rapamycin is associated with less Akt1-mTORC1 signaling, in both SMC and macrophages, during AVF remodeling.

**Figure 4:**
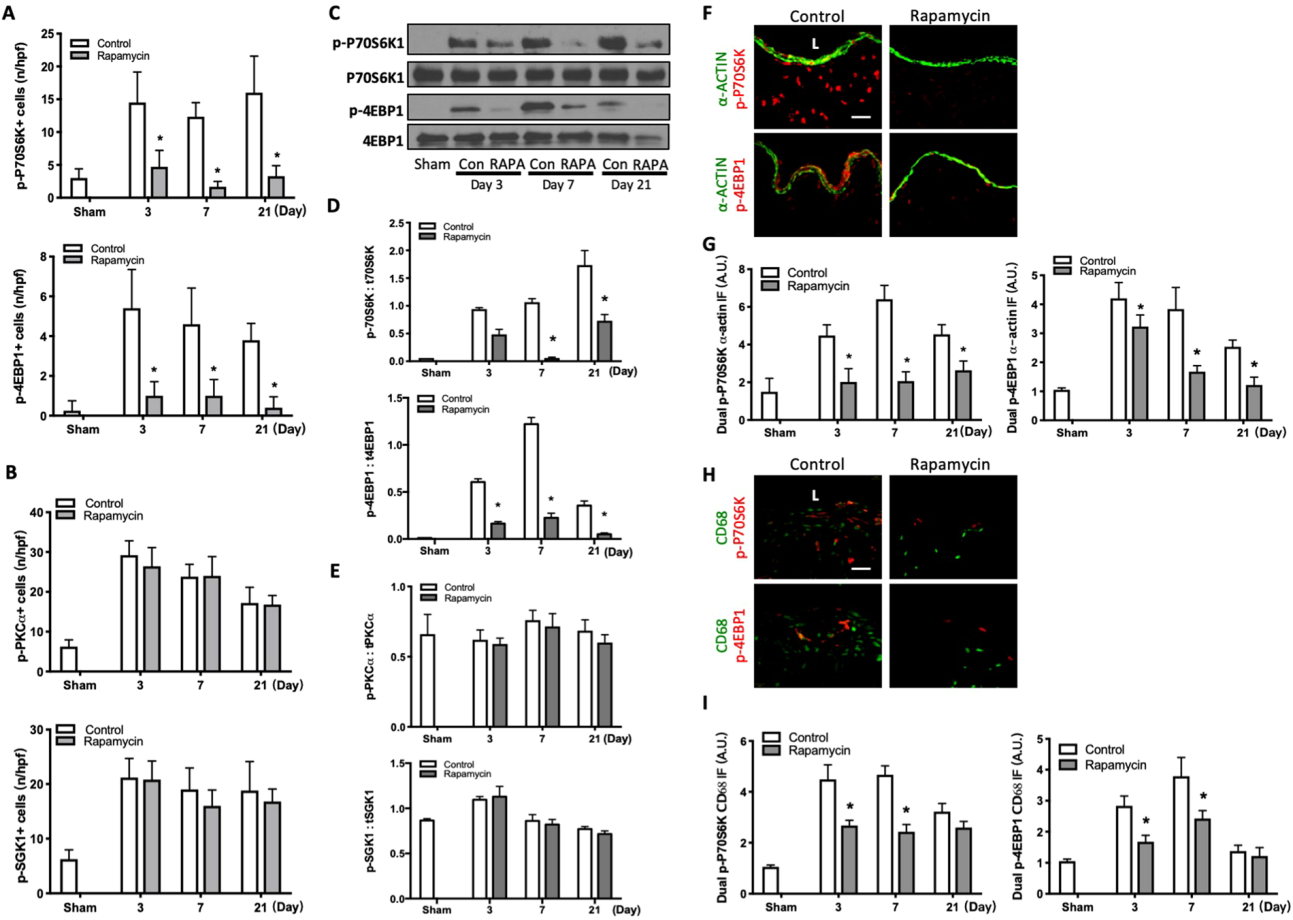
Reduced p70S6K1 and 4EBP1, but not PKCα or SGK1, phosphorylation with rapamycin. (**A**) Bar graphs showing number of p-p70S6K1+ and p-4EBP1+ cells in AVF after rapamycin and control treatment. Top graph, p-p70S6K1: p < 0.0001 (ANOVA). *p < 0.0001, day 3; *P < 0.0001, day 7; *P < 0.0001 at day 21 (post hoc); n = 4–6. Bottom graph, p-4EBP1: p < 0.0001 (ANOVA). *p < 0.0001, day 3; *p < 0.0001, day 7; *p = 0.0010, day 21 (post hoc); n = 5–7. (**B**) Bar graphs showing number of cells in AVF after control vs rapamycin treatment. Top graph, p-PKCα: p = 0.5130 (ANOVA); n = 5. Bottom graph, p-SGK1: p = 0.2569 (ANOVA); n = 4–5. (**C**) Representative Western blot showing p70S6K1 and 4EBP1 phosphorylation after control vs rapamycin treatment. (**D**) Graphs showing densitometry measurement of p70S6K1 and p-4EBP1 phosphorylation. p-p70S6K1: t p70S6K1, p < 0.0001 (ANOVA). *p = 0.0024, day 7; *p = 0.0024, day 21 (post hoc). n = 3. p-4EBP1: t4EBP1, P < 0.0001 (ANOVA). *p = 0.0007, day 3; *p < 0.0001, day 7; *p = 0.0053, day 21 (post hoc). n = 3. (**E**) Graphs showing densitometry measurement of PKCα and SGK1 phosphorylation. p-PKCα: tPKCα, p = 0.9280 (ANOVA); n = 3. p-SGK1: tSGK1, p = 0.6075 (ANOVA). n = 3. (**F**) Photomicrographs of representative IF for α-actin (green) and p-P70S6K1 (red, top row) or p-4EBP1 (red, bottom row) in AVF after control or rapamycin treatment; day 7. (**G**) Bar graphs showing quantification of dual IF after control or rapamycin treatment, normalized to sham vessels. p-P70S6K1-α-actin: p < 0.0001 (ANOVA); *p = 0.0002, day 3; *p < 0.0001, day 7; *p = 0.0030, day 21 (post hoc); n = 4–5. p-4EBP1-α-actin: p < 0.0001 (ANOVA); *p = 0.0378, day 3; *p < 0.0001, day 7; *p = 0.0109, day 21 (post hoc); n = 4–5. (**H**) Representative photomicrographs of IF images for CD68 (green) and p-P70S6K1 (red, top row) or p-4EBP1 (red, bottom row) in AVF after control or rapamycin treatment (day 7). (**I**) Bar graphs showing quantification of dual IF after control or rapamycin treatment, normalized to sham vessels. p-P70S6K1-CD68: p < 0.0001 (ANOVA); *p < 0.0001, day 3; *p < 0.0001, day 7 (post hoc); n = 4–5. p-4EBP1-CD68: p < 0.0001 (ANOVA); *p < 0.0001, day 3; *p < 0.0001, day 7 (post hoc); n = 4–5. Data represent mean ± SEM.

Since these data show that rapamycin reduces mTORC1, but not mTORC2, phosphorylation (Fig. 3), we evaluated the phosphorylation of P70S6K and 4EBP1, downstream targets of mTORC1^23^. There were significantly fewer number of cells that were immunoreactive for p-P70S6K1 or p-4EBP1 in the AVF of mice treated with rapamycin compared to control (Fig. 4A; Supplemental Fig. 4A); however, there was no effect on the number of cells that were immunoreactive for p-PKCα or p-SGK1, downstream targets of mTORC2 (Fig. 4B; Supplemental Fig. 4B). Similarly, AVF treated with rapamycin had significantly decreased expression of phosphorylated P70S6K and 4EBP1 (Fig. 4C,D), but no significant change in expression of phosphorylated PKCα or SGK1 (Fig. 4E; Supplemental Fig. 4C). These results suggest that rapamycin regulates the mTORC1, but not mTORC2 pathway, during venous remodeling.

The AVF of mice treated with rapamycin similarly showed decreased immunoreactivity of p-P70S6K-α-actin dual-positive cells and p-4EBP1-α-actin dual-positive cells (Fig. 4F,G; Supplemental Fig. 4D); rapamycin-treated AVF also showed decreased immunoreactivity of p-P70S6K-CD68 dual-positive cells and p-4EBP1-CD68 dual-positive cells (Fig. 4H,I; Supplemental Fig. 4E). However, there was no difference in immunoreactivity of p-PKCα-α-actin dual-positive cells or p-SGK1-α-actin dual positive cells with rapamycin or control treatments (Supplemental Fig. 4F,G); there was also no difference in immunoreactivity of p-PKCα-CD68 dual-positive cells or p-SGK1-CD68 dual positive cells (Supplemental Fig. 4H,I). These results show that rapamycin is associated with less Akt1-mTORC1, but not mTORC2, signaling in SMC and macrophages, during AVF remodeling.

**Figure 5:**
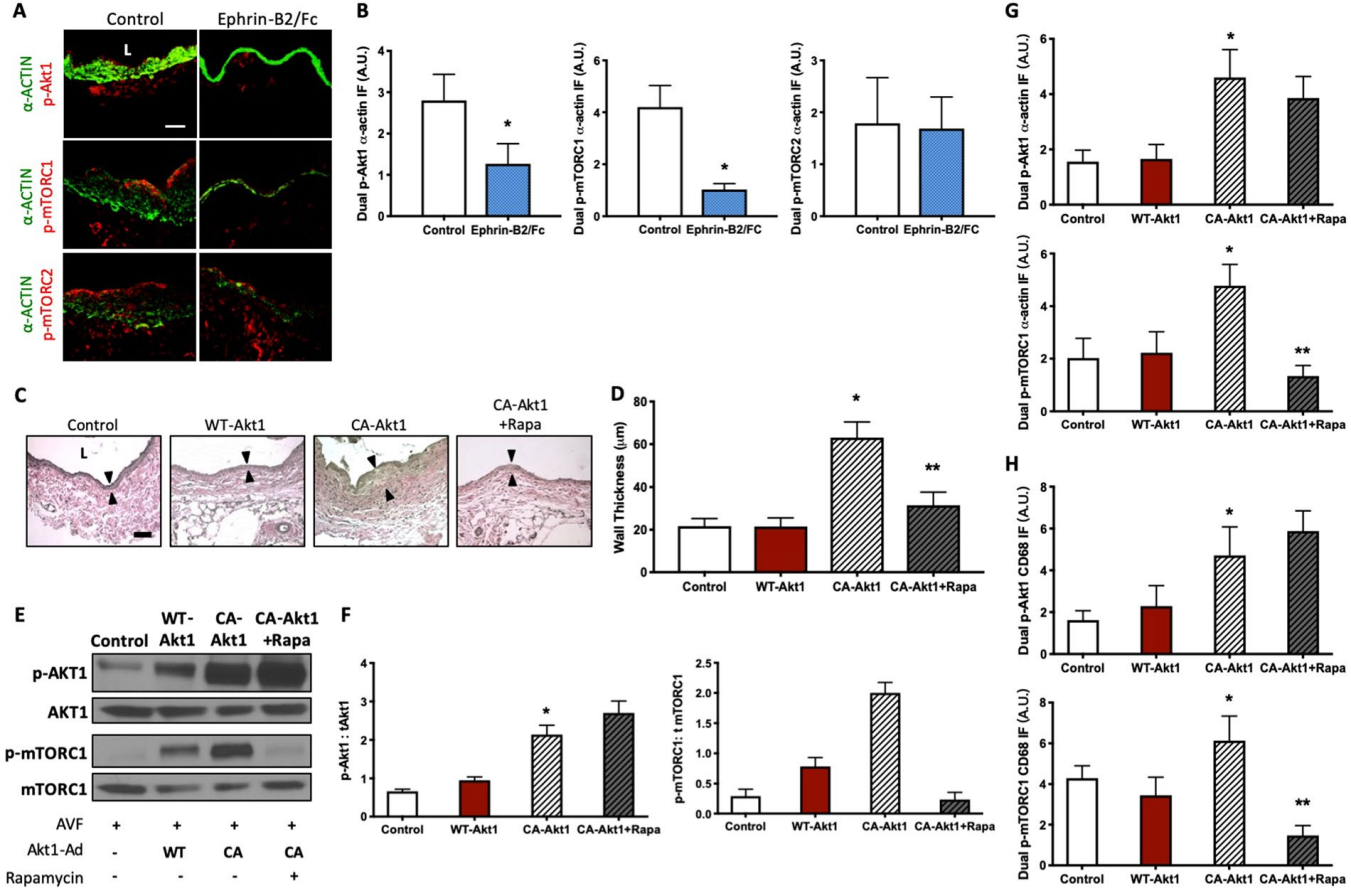
Rapamycin inhibits mTORC1 phosphorylation during venous remodeling. (**A**) Photomicrographs of representative dual p-Akt1-α-actin (top), p-mTORC1-α-actin (middle) and p-mTORC2-α-actin (bottom) IF in control or Ephrin-B2/Fc treated mice AVF (day 21). Scale bar, 25μm. L, lumen. (**B**) Bar graphs showing quantification of dual p-Akt1-α-actin, p-mTORC1-α-actin, and p-mTORC2-α-actin IF after control or Ephrin-B2/Fc treatment. p-Akt1-α-actin: *p = 0.0027 (t-test). p-mTORC1-α-actin: *p < 0.0001 (t-test). p-mTORC2-α-actin, p = 0.8342 (t-test). n = 4–5. (**C**) Photomicrographs showing representative AVF wall thickness in mice treated with control, Ad-WT-Akt1, Ad-CA-Akt1, and Ad-CA-Akt1 with daily 250 μg IP rapamycin injection (day 21). Arrowheads denote vessel wall thickness. Scale bar, 25 µm. L, lumen. (**D**) Bar graph showing AVF wall thickness in mice treated with pluronic gel containing control, WT-Akt1, constitutively active (CA-) Akt1, and CA-Akt1 with 250 μg rapamycin (day 21), p < 0.0001 (ANOVA); control vs WT-Akt1: p > 0.9999; control vs CA-Akt1: *p < 0.0001; Control vs. CA-Akt1 + Rapa: p = 0.0789; WT-Akt1 vs. CA-Akt1: *p < 0.0001; WT-Akt1 vs. CA-Akt1 + Rapa: p = 0.0944; CA-Akt1 vs. CA-Akt1 + Rapa: *p < 0.0001 (post-hoc). n = 4–5. (**E**) Representative Western blot showing expression level of Akt1, p-Akt1, mTORC1 and p-mTORC1 in AVF treated with control, Ad-WT-Akt1, Ad-CA-Akt1, and Ad-CA-Akt1 with rapamycin. (**F**) Graphs showing densitometry measurement of Akt1 and mTORC1 phosphorylation. p-Akt1: t Akt1: p = 0.0015 (ANOVA); Control vs. WT-Akt1: p = 0.5435; Control vs. CA-Akt1: *p = 0.0066; Control vs. CA-Akt1 + Rapa: *p = 0.0019; WT-Akt1 vs. CA-Akt1: *p = 0.0147; WT-Akt1 vs. CA-Akt1 + Rapa: *p = 0.0035; CA-Akt1 vs. CA-Akt1 + Rapa: p = 0.1536 (post hoc); n = 3. p-mTORC1: tmTORC1, P = 0.0025 (ANOVA). Control vs. WT-Akt1: p = 0.8142; Control vs. CA-Akt1: *p = 0.0076; Control vs. CA-Akt1 + Rapa: p = 0.1209; WT-Akt1 vs. CA-Akt1: *p = 0.0125; WT-Akt1 vs. CA-Akt1 + Rapa: p = 0.0566; CA-Akt1 vs. CA-Akt1 + Rapa: *p = 0.0019 (post hoc). n = 3. (**G**) Bar graphs showing quantification of dual IF after delivery of control, Ad-WT-Akt1, Ad-CA-Akt1, and Ad-CA-Akt1 + rapamycin. p-Akt1-α-actin: p < 0.0001 (ANOVA); control vs. CA-Akt1: *p = 0.0001; control vs. CA-Akt1 + Rapa: p = 0.0015; WT-Akt1 vs. CA-Akt1: *p = 0.0003; WT-Akt1 vs. CA-Akt1 + Rapa: p = 0.0033 (post hoc). n = 4–5. p-mTORC1-α-actin: p < 0.0001 (ANOVA); control vs. CA-Akt1: *p = 0.0007; WT-Akt1 vs. CA-Akt1: p = 0.0013; CA-Akt1 vs. CA-Akt1 + Rapa: **p < 0.0001 (post hoc); n = 4–5. (**H**) Bar graphs showing quantification of dual IF after local delivery of control, WT-Akt1, constitutively active CA-Akt1, and CA-Akt1 with rapamycin. p-Akt1-CD68: p = 0.4265 (ANOVA); control vs. CA-Akt1: *p = 0.0041; control vs. CA-Akt1 + Rapa: p = 0.0003; WT-Akt1 vs. CA-Akt1: p = 0.0214; WT-Akt1 vs. CA-Akt1 + Rapa: *p = 0.0013 (post hoc); n = 4–5. p-mTORC1-CD68: p = 0.4662 (ANOVA). control vs. CA-Akt1: *p = 0.0422; control vs. CA-Akt1 + Rapa: p = 0.0025; WT-Akt1 vs. CA-Akt1: p = 0.0036; WT-Akt1 vs. CA-Akt1 + Rapa: p = 0.0287; CA-Akt1 vs. CA-Akt1 + Rapa: **p < 0.0001 (post hoc); n = 4–5. Data represent mean ± SEM.

Since rapamycin inhibits both wall thickness as well as Akt1 and mTORC1 phosphorylation in SMC and macrophages during AVF maturation, we next determined if the Akt1-mTORC1 axis regulates AVF remodeling. We previously showed that Eph-B4 activation with Ephrin-B2/Fc inhibits Akt1 function *in vivo* during venous remodeling^10^; accordingly, we used Ephrin-B2/Fc to inhibit the Akt1-mTORC1 axis. As expected, Ephrin-B2/Fc decreased immunoreactivity of p-Akt1-α-actin dual-positive cells; Ephrin-B2/Fc also diminished p-mTORC1-α-actin dual-positive cells, but not mTORC2-α-actin dual-positive cells, in the absence of rapamycin (Fig. 5A,B). These data suggest that diminished Akt1 activity reduces mTORC1 phosphorylation during venous remodeling.

We next examined whether increased Akt1 activity is associated with increased mTORC1 phosphorylation *in vivo* during AVF maturation. At the time of AVF creation, either control vehicle, wild type (WT)-Akt1 adenovirus (Ad), or constitutively active (CA)-Akt1 adenovirus was placed in pluronic gel on the adventitia of the AVF; viral vectors were found within the EC, SMC, and macrophages in the AVF wall, and both viral vectors had similarly high rates of efficiency (Supplemental Fig. 5A,B). AVF treated with Ad-CA-Akt1 showed increased venous wall thickening compared to AVF treated with control or Ad-WT-Akt1 (Fig. 5C,D). AVF treated with control or Ad-WT-Akt1 showed similar outward remodeling (Supplemental Fig. 5C**)**. Daily IP injections of rapamycin attenuated the increase in wall thickening in AVF treated with Ad-CA-Akt1 (Fig. 5C,D). Similarly, there was increased phosphorylation of Akt1 and mTORC1 in AVF treated with Ad-CA-Akt1, compared to those treated with Ad-WT-Akt1 or control, and rapamycin attenuated phosphorylation of mTORC1, but not Akt1, in AVF treated with CA-Akt1 (Fig. 5E,F). In mice treated with rapamycin, there was no sign of clinical toxicity or significant differences in weight change at day 21 compared to the control group (Supplemental Fig. 5D).

Since rapamycin is associated with reduced mTORC1 phosphorylation in the wall of the remodeling AVF (Figs 3C,D and 5E,F), we next determined whether the inhibitory effects of rapamycin were present in either SMC or macrophages. As expected, there was increased immunoreactivity of p-Akt1-α-actin dual-positive cells and p-mTORC1-α-actin dual-positive cells in AVF treated with Ad-CA-Akt1 compared to control or Ad-WT-Akt1. Rapamycin reduced the immunoreactivity of p-mTORC1-α-actin dual-positive cells, but not p-Akt1-α-actin dual-positive cells, in the AVF treated with Ad-CA-Akt1 (Fig. 5G; Supplemental Fig. 5E). Similarly, rapamycin reduced the immunoreactivity of p-mTORC1-CD68 dual-positive cells, but not p-Akt1-CD68 dual-positive cells, in the AVF treated with Ad-CA-Akt1 (Fig. 5H**;** Supplemental Fig. 5F). These results suggest that rapamycin inhibits mTORC1 signaling in both SMC and macrophages during AVF remodeling.

### Macrophage depletion is associated with reduced AVF wall thickness and patency

**Figure 6:**
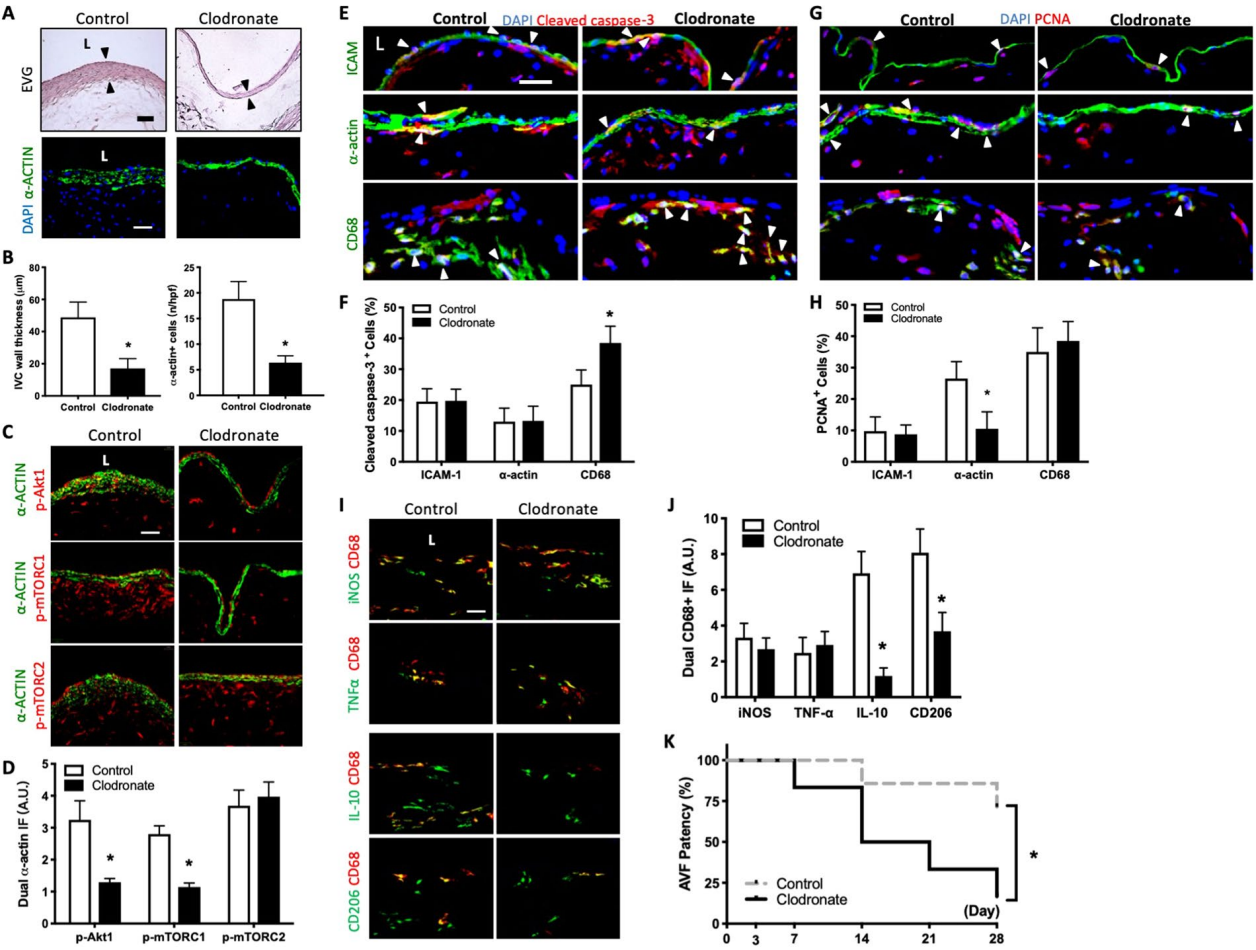
Macrophage depletion is associated with reduced AVF wall thickness and patency. (**A**) Representative photomicrographs showing AVF wall thickness and number of α-actin+ cells in mice treated with clodronate vs. control (day 21). Scale bar, 25 µm. L, lumen (**B**) Bar graphs showing AVF wall thickness (left) and number of α-actin+ cells (right) in AVF after control or clodronate treatment; *p = 0.0005 (t test); n = 5. α-actin+ cell number: *p < 0.0001 (t test); n = 5. (**C**) Representative photomicrographs showing dual IF for a-actin (green) and p-Akt1 (red, first row), p-mTORC1 (red, second row) or p-mTORC2 (red, third row) in AVF after control or clodronate treatment (day 21). (**D**) Bar graph showing quantification of dual IF in AVF after control or clodronate treatment. p-Akt1-α-actin: *p < 0.0001 (t test); n = 5. p-mTORC1-α-actin: *p = 0.0011 (t test); n = 5. p-mTORC2-α-actin: p = 0.5549 (t test); n = 5. (**E**) Photomicrographs showing representative IF of cleaved caspase-3 (red) merged with ICAM, α-actin or CD68 (green), and DAPI (blue) in AVF of control or clodronate treated mice (day 7); L, lumen; scale bar, 25 μm. White arrowheads indicate merged signal. (**F**) Bar graphs showing percentage of dual positive cells (day 21). Cleaved caspase-3-ICAM: p > 0.9999 (t test); n = 4–5. Cleaved caspase-3-α-actin: p = 0.9315 (t test). n = 4–5. Cleaved caspase-3-CD68: *p = 0.0027 (t test). n = 4–5. (**G**) Photomicrographs showing representative IF of PCNA (red) merged with ICAM, α-actin or CD68 (green), and DAPI (blue) in AVF of control or clodronate treated mice (day 7); L, lumen; scale bar, 25 μm. White arrowheads indicate merged signal. (**H**) Bar graph showing percentage of dual positive cells (day 21). PCNA-ICAM positive cells: p = 0.8139 (t test); PCNA-α-actin: *P = 0.0035 (t test); PCNA-CD68: p = 0.8547 (t test). n = 4–5. (**I**) Representative photomicrographs showing dual IF for CD68 (red) and iNOS (green, top row), TNF-α (green, second row), IL-10 (green, third row) or CD206 (green, bottom row) in AVF after control or clodronate treatment; day 21. Scale bar, 25 μm. L, lumen. (**J**) Bar graphs showing quantification of dual IF after control or clodronate treatment (day 21). CD68-iNOS: p = 0.7311 (t test). CD68-TNF-α: p < 0.8422 (t test). CD68-IL-10: p < 0.0001 (t test). CD68-CD206: p = 0.0006 (t test). n = 5. (**K**) Line graph showing AVF patency rate in mice treated with control or clodronate IP injections. *P = 0. 0.0372 (Log-rank), n = 6–7 in each arm.

We have previously shown that M2-type macrophages play a role during venous remodeling such as occurs during vein graft adaptation^24^ and AVF maturation^25^; delivery of MCP-1 to the AVF adventitia increased M2-type macrophages and increased AVF wall thickness^25^. Since our data suggest that rapamycin has an effect on macrophage proliferation (Fig. 1), M1 and M2 marker expression (Fig. 2), and Akt1-mTORC1 signaling (Figs 3–5**)**, we next examined whether depletion of macrophages would improve AVF patency. After IP injections of clodronate-containing liposomes, there were significantly reduced numbers of CD68 immunoreactive cells in the AVF wall (Supplemental Fig. 6A). Macrophage depletion was associated with reduced wall thickening that was characterized by fewer α-actin immunoreactive cells (day 21; Fig. 6A,B). There was also reduced immunoreactivity of p-Akt1-α-actin dual-positive cells and p-mTORC1-α-actin dual-positive cells, but no change in p-mTORC2-α-actin dual-positive cells, in macrophage-depleted AVF compared to control (Fig. 6C,D). Clodronate increased the number of apoptotic macrophages but had no effect on EC or SMC apoptosis (Fig. 6E,F); there was no compensatory increase in proliferation in any cell type (Fig. 6G,H**)**. At day 7, clodronate-treated AVF showed reduced immunoreactivity of CD68-iNOS, CD68-TNF-α, CD68-IL-10 and CD68-CD206 dual-positive cells in the adventitia compared with control AVF (Supplemental Fig. 6B). However, at day 21, there was little immunoreactivity of CD68-iNOS dual-positive cells or CD68-TNF-α dual-positive cells in either control AVF or clodronate-treated AVF; interestingly, control AVF had some immunoreactivity of CD68-IL-10 dual-positive cells and CD68-CD206 dual-positive cells that were not observed with clodronate treatment (Fig. 6I,J). Because macrophage depletion was associated with reduced AVF wall thickness, we next assessed whether the reduced number of macrophages was also associated with altered AVF patency. Macrophage depletion significantly reduced the AVF patency by day 28 (Fig. 6K). These data are consistent with clodronate depletion of both M1- and M2-type macrophages during AVF maturation and suggest a mechanistic role for macrophages during AVF adaptive remodeling.

### Rapamycin treatment is associated with reduced AVF wall thickness but increased AVF patency

**Figure 7:**
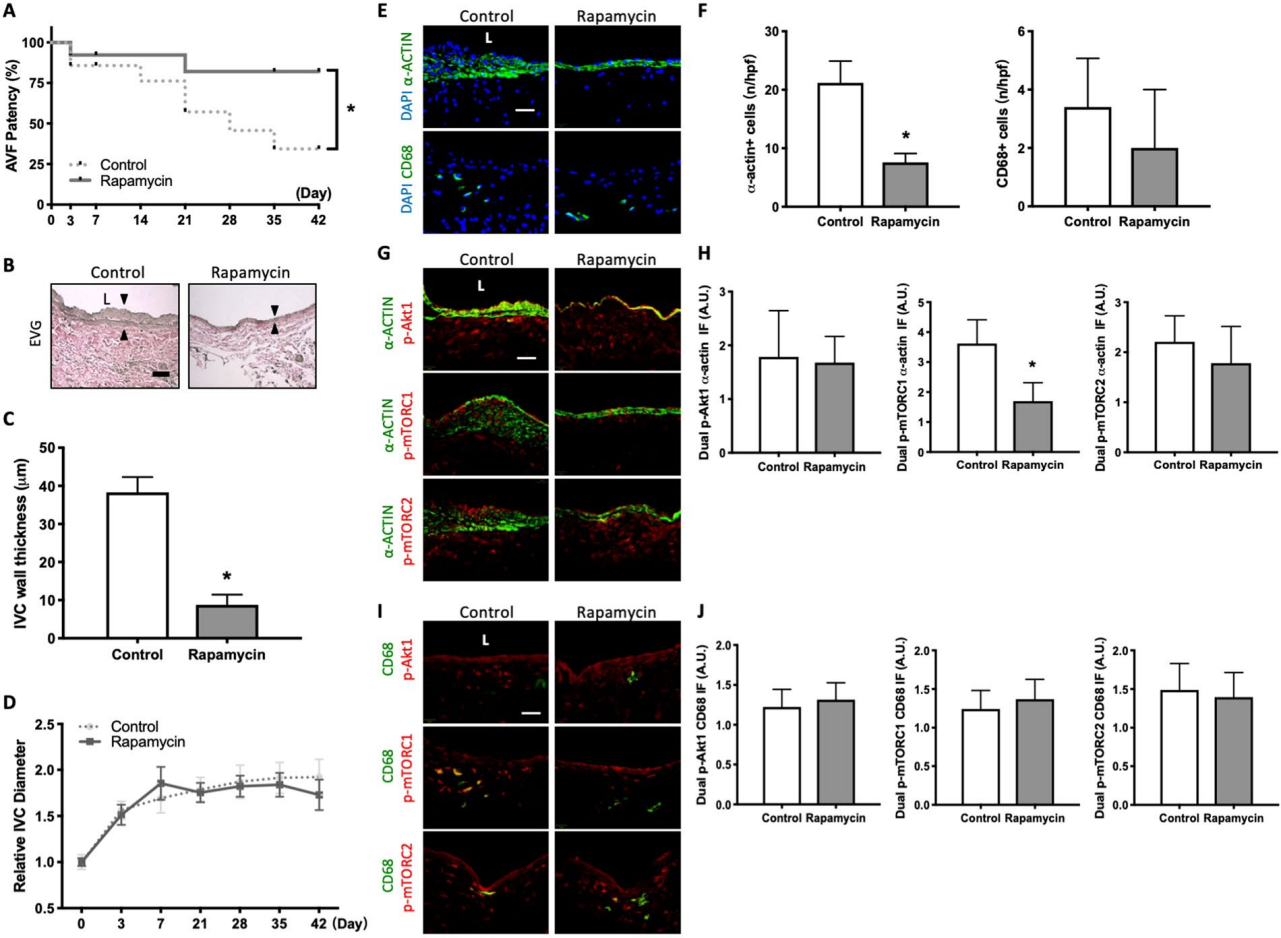
Rapamycin treatment is associated with reduced AVF wall thickness but increased AVF patency. (**A**) Line graph showing AVF patency rate in mice treated with control vs rapamycin IP injections. *P = 0.0495 (Log-rank), n = 13–14 in each arm. (**B**) Representative photomicrographs showing AVF wall thickness in mice treated with control or rapamycin (day 42). Arrowheads denote wall thickness. Scale bar, 25μm. L, lumen. (**C**) Bar graph showing AVF wall thickness in after control or rapamycin treatment (Day 42); *p < 0.0001 (t test). n = 5. (**D**) Line graph showing relative AVF diameter in mice treated with control or rapamycin, normalized to day 0; p = 0.2603 (ANOVA); n = 6–8. (**E**) Photomicrographs of represen tative IF of α-actin+ (top row) and CD68+ cells (bottom row) in control or rapamycin treated mice AVF (day 42). (**F**) Bar graphs quantifying number of α-actin+ (left) and CD68+ cells (right) in AVF after control or rapamycin treatment; α-actin: *p < 0.0001 (t-test); CD68: p = 0.2643 (t test); day 42. n = 5. (**G**) Photomicrographs of representative dual IF of a-actin (green) and p-Akt1 (red, first row), p-mTORC1 (red, second row) or p-mTORC2 (red, third row) in AVF after control or rapamycin treatment (day 42). (**H**) Bar graphs showing quantification of dual IF in AVF after control or rapamycin treatment (day 42). p-Akt1-α-actin: p = 0.8126 (t test); p-mTORC1-α-actin: *p = 0.0026 (t test). p-mTORC2-α-actin: p = 0.3206 (t test); n = 5. (**I**) Photomicrographs of representative dual IF for CD68 (green) and p-Akt1 (red, first row), p-mTORC1 (red, second row) or p-mTORC2 (red, third row) in AVF after control or rapamycin treatment (day 42). (**J**) Bar graphs showing quantification of dual IF in AVF after control or rapamycin treatment (Day 42). p-Akt1-CD68: p = 0.5195 (t test). p-mTORC1-CD68: p = 0.4453 (t test). p-mTORC2-CD68: p = 0.6633 (t test); n = 5.

The mouse AVF model is characterized by increased wall thickness and dilation between days 0 and 28, mimicking human AVF maturation; however, between days 28 and 42 there is increased neointimal hyperplasia and loss of patency in approximately 1/3 of mice, mimicking human AVF late failure^26^. Since rapamycin treatment was associated with reduced AVF wall thickness and attenuated SMC and macrophage proliferation (Fig. 1), we determined the effects of rapamycin on AVF patency; daily rapamycin or control vehicle injection was continued up to postoperative day 42. In mice treated with rapamycin daily, there was no sign of clinical toxicity or significant differences in weight change compared to control mice (Supplemental Fig. 7A); there was also no difference in the technical success rate of AVF creation in rapamycin treated mice compared to control mice (Supplemental Fig. 7B). Rapamycin-treated mice showed improved AVF patency by day 42 (Fig. 7A); there was no significant difference in AVF patency if rapamycin was delivered directly to the adventitia in a single dose at the time of AVF creation (Supplemental Fig. 7B). Mice treated with IP injections of rapamycin had AVF that showed less thickening but a similar rate of dilation compared to control mice (Fig. 7B–D).

At day 42, rapamycin-treated AVF showed fewer number of α-actin immunoreactive cells, with no change in the number of CD68 immunoreactive cells, compared with control AVF (Fig. 7E,F). AVF of rapamycin treated mice showed reduced immunoreactivity of α-actin-mTORC-1 dual-positive cells without any change in immunoreactivity of α-actin-p-Akt1 dual-positive cells or α-actin-p-mTORC2 dual-positive cells (Fig. 7G,H). However, AVF of rapamycin treated mice had similar immunoreactivity of p-Akt1-CD68 dual-positive cells, p-mTORC1-CD68 dual-positive cells and p-mTORC2-CD68 dual-positive cells compared to control (Fig. 7I,J). In toto, these data suggest that rapamycin has sustained inhibition of mTORC1 activity in SMC, reducing wall thickness and improving AVF patency.

### Rapamycin enhances early AVF remodeling to improve patency

**Figure 8:**
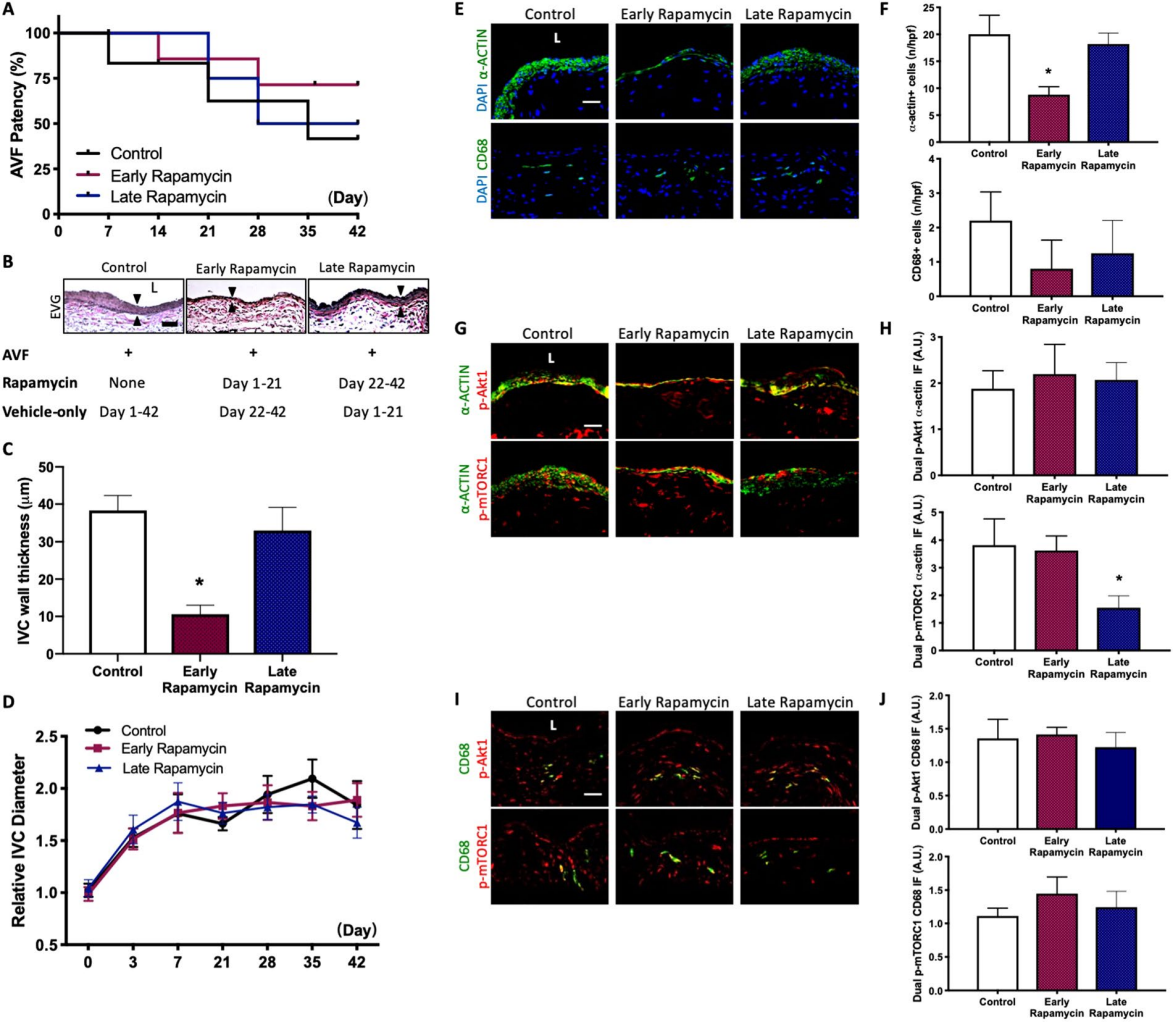
Rapamycin enhanced early AVF remodeling to improve patency. (**A**) Line graph showing AVF patency rate in mice treated with control, early vs late rapamycin. Control vs early rapamycin: P = 0.0591 (Log-rank); control vs late rapamycin: P = 0.812 (Log-rank); n = 5–6 in each group. (**B**) Representative photomicrographs showing AVF thickness in mice treated with control, early rapamycin or late rapamycin (day 42). Arrowheads denote wall thickness. (**C**) Bar graph showing AVF wall thickness in after control, early rapamycin or late rapamycin treatment (Day 42); p < 0.0001 (ANOVA); control vs early rapamycin: p < 0.0001 (ANOVA); n = 5. (**D**) Line graph showing relative AVF diameter in mice treated with control, early rapamycin or late rapamycin, normalized to day 0; p = 0.6767 (ANOVA); n = 5–6. (**E**) Photomicrographs of representative IF of α-actin+ (top row) and CD68+ cells (bottom row) in control, early or late rapamycin treated AVF (day 42). (**F**) Bar graphs quantifying number of α-actin+ and CD68+ cells in AVF after control, early rapamycin or late rapamycin treatment; α-actin: p < 0.0001 (ANOVA); *p < 0.0001, control vs early rapamycin; CD68: p = 0.0813 (ANOVA); day 42. n = 4–5. (**G**) Photomicrographs of representative dual IF of a-actin (green) and p-Akt1 (red, first row) or p-mTORC1 (red, second row) in AVF after control, early or late rapamycin treatment (day 42). (**H**) Bar graphs showing quantification of dual IF in AVF after control, early rapamycin or late rapamycin treatment (day 42); p-Akt1-α-actin: p = 0.6067 (ANOVA); p-mTORC1-α-actin: *p = 0.0003 (ANOVA); control vs late rapamycin: p = 0.009; n = 5. (**I**) Photomicrographs of representative dual IF for CD68 (green) and p-Akt1 (red) or p-mTORC1 (red) in AVF after control, early rapamycin or late rapamycin treatment (day 42). (**J**) Bar graphs showing quantification of dual IF in AVF after control, early rapamycin or late rapamycin treatment (Day 42). p-Akt1-CD68: p = 0.4474 (ANOVA); p-mTORC1-CD68: p = 0.181 (ANOVA); n = 5.

To determine whether the increased patency rate observed after rapamycin treatment is due to enhancement of AVF remodeling during the early maturation phase or due to reduced neointimal hyperplasia during later remodeling, rapamycin treatment was given either only from day 1–21 (early rapamycin) or only from day 22–42 (late rapamycin); control AVF received only vehicle injections from day 1–42. Compared to control mice, mice treated with early rapamycin treatment had a trend towards improved AVF patency by day 42; however, compared to control mice, there was no significant improvement in AVF patency with late rapamycin treatment (Fig. 8A). Mice treated with early rapamycin, but not late rapamycin, had AVF that showed reduced thickening, compared to control mice (Fig. 8B,C). Mice treated with early rapamycin and late rapamycin had a similar rate of dilation compared to control mice (Fig. 8D).

At day 42, AVF treated with early rapamycin, but not AVF treated with late rapamycin, showed fewer number of α-actin immunoreactive cells, compared to control AVF (Fig. 8E,F). However, AVF treated with control, early rapamycin or late rapamycin showed no difference in the number of CD68 immunoreactive cells (Fig. 8E,F). AVF treated with control, early rapamycin or late rapamycin also showed similar immunoreactivity of α-actin-p-Akt1 dual-positive cells (Fig. 8G,H). AVF treated with late rapamycin, but not AVF treated with early rapamycin, had reduced immunoreactivity of α-actin-p-mTORC1 dual-positive cells compared to control (Fig. 8G,H). AVF treated with control, early rapamycin, or late rapamycin had similar immunoreactivity of p-Akt1-CD68 dual-positive cells and p-mTORC1-CD68 dual-positive cells (Fig. 8I,J). These data suggest that rapamycin improves AVF patency by enhancing AVF remodeling during the early phase of maturation, whereas rapamycin treatment only during later remodeling does not improve patency or reduce wall thickening.

## Discussion

This study shows that rapamycin reduces wall thickening and early inflammation in AVF as well as proliferation in SMC and macrophages (Fig. 1), suppressing both M1 and M2 macrophage subtypes (Fig. 2). Rapamycin also inhibits Akt1-mTORC1 phosphorylation and downstream signaling in both SMC and macrophages during early AVF remodeling (Figs 3 and 4). Macrophage depletion with clodronate reduces wall thickening but is accompanied by reduced AVF patency with reduced numbers of M1- and M2-type macrophages (Fig. 6). However, rapamycin leads to persistently reduced AVF wall thickening and improved patency by enhancing AVF remodeling during the early phase of remodeling (Figs 7 and 8). These results show that rapamycin improves AVF remodeling and long-term patency by reducing inflammation and cell proliferation during early maturation; in addition, macrophages are necessary for adaptive venous remodeling.

Our primary finding is that rapamycin improves AVF patency while reducing wall thickening during the early phase of maturation, with no effect on AVF dilation. Given the need for therapies that improve vascular access patency, rapamycin and other antiproliferative agents are currently being investigated in clinical studies. A recent clinical trial studied a rapamycin-eluting collagen membrane in 12 patients and showed minimal toxicity, and 1-year primary patency rate of 76% with the treatment, highlighting a significant improvement in AVF matruation^27^. There are currently 2 clinical trials investigating the use of rapamycin to improve AVF patency. In the ACCESS trial (NCT02513303)^28^, patients in the treatment group receive a single dose of rapamycin delivered locally, via collagen implants, to the vessel wall at the time of AVF creation. In the SAVE trial (NCT01595841)^29^, patients requiring angioplasty to treat AVF failure are randomized to receive either rapamycin or placebo. Although these trials are still in progress, there are no pre-clinical studies examining the effects of rapamycin on AVF patency. Our data suggests that rapamycin treatment initiated during early maturation reduces both SMC and macrophages in the AVF wall (Figs 1, 7 and 8), contributing to improved AVF patency, and support the hypothesis of the ACCESS trial. It is possible that differences between our mouse model and human AVF are important; however, the mouse model recapitulates human AVF maturation as well as failure rates, suggesting its utility in understanding human physiology^14,26^. Moreover, mTOR plays a central role in regulating metabolic cell processes, including protein and lipid synthesis, and autophagy. Chronic mTORC1 inhibition has been associated with muscle atrophy, reduced adipogenesis, decreased pancreatic β-cell proliferation and increased ketogenesis^30^; however, despite these potential side effects associated inhibition of mTORC1, the daily 1.4–1.5 μg/cm^2^ dose of rapamycin used in our study did not affect AVF maturation or cause any clinical toxicity.

Our data show that during early AVF remodeling, rapamycin treatment is associated with reduced SMC proliferation and mTORC1 signaling but has no effect on proliferation and mTOR signaling in EC (Figs 1 and 3). These results are consistent with our previous work showing that selective knockdown of Akt1 from SMC, but not EC, abolishes AVF remodeling^10^, and are also in agreement with the long-established role of SMC during vascular remodeling. There may be a dual function of mature SMC in AVF, with differentiated SMC contributing to medial wall thickening and resultant venous maturation, and dedifferentiated SMC contributing to detrimental neointimal hyperplasia^18^. It has been suggested that neointimal hyperplasia and the resulting thrombosis are the major pathological etiologies of AVF failure^31^. Rapamycin most likely reduces the inflammation that causes SMC proliferation in AVF, but not SMC proliferation directly^32^, as shown by its lack of effect on SMC number when given during late remodeling (Fig. 8). Although rapamycin treatment during late AVF remodeling reduces mTORC1 signaling in SMC, it does not improve patency or reduce wall thickening (Fig. 8). This observation confirms that the increased patency rate with rapamycin treatment is due to enhancement of AVF remodeling during the early maturation phase when inflammation is most significant (Figs 1 and 2). The exact implications of improved patency with a thinner wall remain to be determined; wall thickening is required for AVF maturation, but uncontrolled pathologic remodeling leads to AVF failure^5,6^. Our data suggests that rapamycin may allow an optimal amount of initial outward remodeling, but appears to prevent the excessive wall thickening and inward remodeling that can lead to AVF failure.

AVF creation is associated with local inflammation^33^ and this inflammatory response involves the recruitment of macrophages, lymphocytes, and upregulation of cytokines such as IL-6 and TNF-α, all of which are associated with fistula failure^20,33,34^. There is mounting evidence that mTORC1-mediated signaling regulates both adaptive and innate immune cell function^35–37^, and more specifically, rapamycin attenuates the inflammatory response following vascular injury, with secondary effects on SMC and EC proliferation^38,39^. Similarly, we observed that rapamycin treatment is associated with reduced number and proliferation of macrophages (Fig. 1) as well as attenuated Akt1-mTORC1 signaling in macrophages during the early maturation phase (Fig. 3). Our data also shows that following macrophage depletion, SMC proliferation decreases. Inflammatory cytokines may directly stimulate SMC proliferation and contribute to wall thickening^40–42^, and Akt activation may promote vascular SMC hypertrophy, leading to formation of neointimal hyperplasia^43^. Reducing macrophage accumulation decreases SMC hyperplasia *in vivo*, suggesting, as we observe in our AVF model, that macrophages play an important role in determining SMC activity during vascular remodeling^40^.

Although the exact role of specific macrophage subtypes during AVF maturation remains unknown, M1 macrophages accumulate during the early maturation phase of venous remodeling, with subsequent increased numbers of M2 macrophages during later maturation phases (Fig. 6J)^19^. Thus, limiting rapamycin delivery to the very early phase of maturation to inhibit M1-type macrophage activity appears to result in similar or even more improved AVF remodeling (Fig. 8). We have previously shown that CD44 promotes accumulation of M2-type macrophages, ECM deposition, and inflammation resulting in enhanced AVF maturation^25^. We have also shown that M2-type macrophage function may be an important mechanism in regulating venous remodeling such as occurs during vein graft adaptation^24^. This study shows that rapamycin attenuates both M1 and M2 macrophage activity. While inhibition of pro-inflammatory M1 activity might be advantageous in improving AVF patency, complete diminution of macrophage function appears to be detrimental to AVF patency, possibly by sustained inhibition of the M2-type macrophages (Fig. 6). However, when used to reduce, as opposed to deplete, both macrophage phenotypes, rapamycin is associated with improved AVF remodeling and patency (Fig. 7). There are mixed reports of rapamycin affecting M2-type macrophage survival and polarizing the phenotype to an M1-like inflammatory response both *in vivo* and *in vitro*^44^ as well as favoring macrophage polarization toward an M2 anti-inflammatory response^45^; nonetheless, rapamycin treatment is associated with reduced M1- and M2-type macrophages during venous remodeling.

In conclusion, rapamycin improves AVF patency and early venous remodeling while reducing wall thickening and early inflammation. These effects are associated with reduced Akt1-mTORC1 signaling in macrophages and SMC during the early maturation phase and sustained reduction in SMC during the late maturation phase. Macrophages are essential for AVF remodeling and M2 macrophages may have a mechanistic role in AVF maturation. The mTORC1 pathway is a key regulator of AVF maturation and its inhibition with rapamycin may be a translational strategy to improve AVF patency.

## Methods

### Study approval

All animal experiments were performed in strict compliance with federal guidelines and with approval from the Yale University IACUC.

### Infrarenal aorto-caval fistula

Mice used for this study were wild type C57BL6/J. Mice were 9–12 weeks of age when the infrarenal aorto-caval fistulae were created as previously described^14,26^; only male mice were studied since female sex is the only predictor of non-maturation of human AVF in some studies^46^. Briefly, AVF were created by needle puncture from the aorta into the inferior vena cava (IVC) using a 25 G needle. Visualization of pulsatile arterial blood flow in the IVC was assessed as a technically successful creation of AVF. Following surgery, all animals were monitored daily and evaluated weekly by a veterinarian for changes in health status.

### Confirmation of fistula patency and measurement of fistula dilation

Doppler ultrasound (40 MHz; Vevo770 High Resolution Imaging System; Visual Sonics Inc., Toronto, Ontario, Canada) was used to confirm the patency of the AVF and to measure the diameter of the vessels as previously described^14,26^. Doppler ultrasound was performed prior to operation (day 0 values) and serially post-operatively. Increased end-diastolic flow through the aorta and a high velocity pulsatile flow within the IVC confirmed the presence of an AVF during post-operative studies. Patency was again confirmed at time of AVF harvest by direct visualization of pulsatile arterial blood flow into the IVC, and in all cases correlated with the ultrasound findings.

### Histology

After euthanasia, the circulatory system was flushed under pressure with PBS followed by 10% formalin and the AVF was harvested en bloc. The tissue was then embedded in paraffin and cut in 5 μm cross sections. Hematoxylin and eosin staining was performed for all samples. Elastin Van Gieson (EVG) staining was used to measure intima-media thickness in 5 μm cross sections of the IVC using sections obtained 100–200 µm cranial to the fistula. Four equidistant points around the IVC and opposite the aortic wall were averaged in each cross section to obtain the mean AVF outer wall thickness. Additional unstained cross sections in this same region were used for immunofluorescence microscopy.

### Immunohistochemistry and Immunofluorescence

Tissue sections were de-paraffined using xylene and a graded series of alcohols. Sections were heated in citric acid buffer (pH 6.0) at 100 °C for 10 min for antigen retrieval. The sections were blocked with 5% bovine serum albumin PBS containing 0.05% Triton X‐100 (T‐PBS) for 1 h at room temperature prior to incubation overnight at 4 °C with the primary antibodies diluted in T-PBS. All the primary antibodies have been listed in the Table 1. Sections were then treated with secondary antibodies at room temperature for 1 h using goat anti-rabbit Alexa Fluor 488 (Life Technologies), donkey anti-goat Alexa-Fluor-488 (Life Technologies), or donkey anti-rabbit Alexa-Fluor-568 (Life Technologies). Sections were stained with Slow Fade® Gold Antifade Mount with DAPI (Life Technologies) and coverslip was applied. Digital fluorescence images were captured and intensity of immunoreactive signal was measured using Image J software (NIH, Bethesda, Maryland). Intensity of the merge signal was determined by applying a color threshold selective for the appropriate signal.

**Table 1:** List of Antibodies.

Target antigen	Vendor or Source	Catalog #
Cleaved caspase-3	Cell Signaling	9664
proliferating cell nuclear antigen	Dako	M0879
Collagen I	Novus Biologicals	NB600-408
Collagen III	Novus Biologicals	NB600-594
fibronectin	Abcam	ab2413
CD68	Bio-Rad	MCA1957
iNOS	Cell Signaling	2977 S
interleukin-10	Abcam	ab9969
TNFα	Abcam	ab9635
CD206	Bio-Rad	MCA2235T
VECAM1	Abcam	ab134047
ICAM1	R&D Systems	AF796-SP
Phospho-Akt1	Cell Signaling	9018
Akt1	Cell Signaling	2967
Phospho-mTOR (Ser2481)	Cell Signaling	2974
Phospho-mTOR (Ser2448)	Cell Signaling	2971
phospho-4EBP1	Cell Signaling	2855
4EBP1	Cell Signaling	9452
phospho-70SK1	Abcam	17464
70S6K1	Cell Signaling	9202
phospho-PKCα	Abcam	23513
PKCα	Cell Signaling	2056
Phospho-SGK1	Thermo Fischer	44-1260 G
SGK1	Abcam	59337
Alpha-actin	Dako	M0851
GAPDH	Cell Signaling	2118

### Western blot

The venous limb of the AVF was harvested and treated with RIPA lysis buffer containing protease inhibitors. Equal amounts of protein were loaded and run in SDS-PAGE followed by Western blot analysis. Protein expression was probed with the antibodies listed in Table 1.

Membranes were developed using Western Lightning Plus ECL reagent (PerkinElmer). Membranes were stripped with Restore Western Blot Stripping Buffer (Pierce Biotechnology) and then re-probed. Band densitometry was performed using ImageJ and was normalized to GAPDH or the ratio of phosphorylated to total protein was calculated.

### Rapamycin and clodronate treatment

Intraperitoneal (IP) injections of rapamycin (100 μg; #553212, Sigma Aldrich) were delivered every 24 h beginning on the day of operation and continued throughout the study period. In mice treated with adenovirus containing constitutively active Akt1, 250 μg of rapamycin was used. The control group received an equal volume injection of vehicle (DMSO) as control. In the adventitial delivery group, pluronic gel was used to deliver 100 μg of rapamycin to the adventitia of the venous AVF wall of at the time of surgery.

Intraperitoneal injections of clodronate-containing liposomes (0.5 mg/Kg; CLD-8909, Encapsula Nano Sciences) were delivered every 72 hr beginning on postoperative day 1 and continued throughout the study period. The control group received an equal volume injection of vehicle (PBS). Intraperitoneal injections of 20 µg Ephrin-B2/Fc (R&D) were delivered 24 h prior to AVF creation and every 48 h thereafter. Control mice received an equal volume injection of vehicle (PBS) as control.

### Adenovirus treatment

Infrarenal aorto-caval AVF were created as described above. After unclamping and confirming fistula flow, 1·10^6^ copies of commercially available vectors (Vector Biolabs, Malvern, PA) containing either constitutively active Akt1 adenovirus (Myr-HA-Akt1), or a control virus (WT-HA-Akt1) were applied to the AVF adventitial surface in a 25% w/v pluronic gel. The HA reporter tag in these vectors were used for immunofluorescent confirmation of virus delivery. After visual confirmation that the pluronic gel mixture had solidified, the abdomen was closed as described above.

### Statistics

Data are represented as mean value ± SEM. All data were analyzed using Prism 8 software (GraphPad Software, Inc., La Jolla, CA). The Shapiro-Wilk test was performed to analyze normality and the F test was performed to evaluate homogeneity of variances. For two-group comparisons with normally distributed data, the unpaired Student’s t test was used for data with equal variances among groups and the unpaired Student’s t test with Welch correction was used for data with unequal variances. For multiple group comparisons with normally distributed data, the one-way ANOVA followed by the Sidak’s post-hoc test was used. Patency outcomes were analyzed with the use of Kaplan–Meier curves to display the distribution of occlusion events detected over time. P values < 0.05 were considered significant.

## Supplementary information

Supplemental Figures

## Data Availability

All data generated or analyzed during this study are included in this published article (and its Supplementary Material files). Other additional data are also available from the corresponding author on reasonable request.
