# Author Correction: Inhibition of the Akt1-mTORC1 Axis Alters Venous Remodeling to Improve Arteriovenous Fistula Patency

**DOI:** 10.1038/s41598-020-65540-w

**Published:** 2020-05-14

**Authors:** Xiangjiang Guo, Arash Fereydooni, Toshihiko Isaji, Jolanta Gorecka, Shirley Liu, Haidi Hu, Shun Ono, Michelle Alozie, Shin Rong Lee, Ryosuke Taniguchi, Bogdan Yatsula, Naiem Nassiri, Lan Zhang, Alan Dardik

**Affiliations:** 1 Vascular Biology and Therapeutics Program, Yale School of Medicine, New Haven, CT USA; 2 Department of Vascular Surgery, Renji Hospital, School of Medicine, Shanghai Jiao Tong University, Shanghai, China; 3 Division of Vascular and Endovascular Surgery, Department of Surgery, Yale School of Medicine, New Haven, CT USA

Correction to: *Scientific Reports* 10.1038/s41598-019-47542-5, published online 30 July 2019

This Article contains an error. The p-mTORC1 control panel in Figure 8G was inadvertently duplicated from the p-Akt1 control panel in Figure 6C. The correct panels for Figure 8G and 8H appear below in Figure 1, as Panels 1A and 1B respectively. The statistical analyses were re-calculated for the m-TORC1 data, and the conclusions were unaffected.

**Figure 1:**
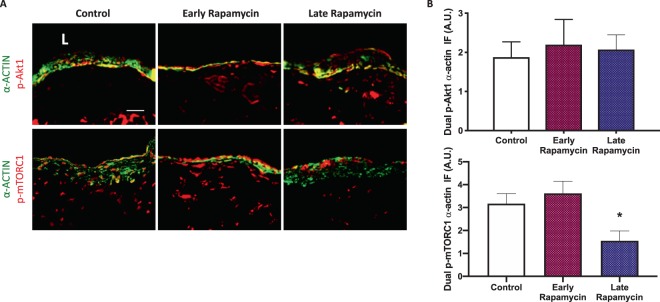
Rapamycin enhanced early AVF remodeling to improve patency. **(A)** Photomicrographs of representative dual IF of a-actin (green) and p-Akt1 (red, first row) or p-mTORC1 (red, second row) in AVF after control, early or late rapamycin treatment (day 42). **(B)** Bar graphs showing quantification of dual IF in AVF after control, early rapamycin or late rapamycin treatment (day 42); p-Akt1-α-actin: p = 0.6067 (ANOVA); p-mTORC1-α-actin: *p = 0.0004 (ANOVA); control vs late rapamycin: p = 0.0016; n=5 for all groups except n = 4 in control group for p-mTORC1-α-actin.

