# Supplementary material for: Inhibition of the Akt1-mTORC1 Axis Alters Venous Remodeling to Improve Arteriovenous Fistula Patency
Source: Sci Rep. 2019 Jul 30;9:11046. doi: 10.1038/s41598-019-47542-5 (PMC6667481; doi:10.1038/s41598-019-47542-5)
Supplement: Supplementary file 1 — Supplemental Figures [file 41598_2019_47542_MOESM1_ESM.pdf]

Supplementary Material

Inhibition of the Akt1-mTORC1 Axis Alters Venous Remodeling to Improve Arteriovenous Fistula Patency

Xiangjiang Guo,*[1,2] Arash Fereydooni,*[1] Toshihiko Isaji,[1] Jolanta Gorecka,[1] Shirley Liu,[1] Haidi Hu,[1] Shun Ono,[1] Michelle Alozie,[1] Shin Rong Lee,[1] Ryosuke Taniguchi,[1] Bogdan Yatsula,[1] Naiem Nassiri,[3] Lan Zhang,[2] Alan Dardik[1, 3]

1. Vascular Biology and Therapeutics Program, Yale School of Medicine, New Haven, CT USA

2. Department of Vascular Surgery, Renji Hospital, Shanghai Jiao Tong University, Shanghai, China

3. Section of Vascular and Endovascular Surgery, Department of Surgery, Yale University School of Medicine, New Haven, CT, USA

* These authors contributed equally to this article.

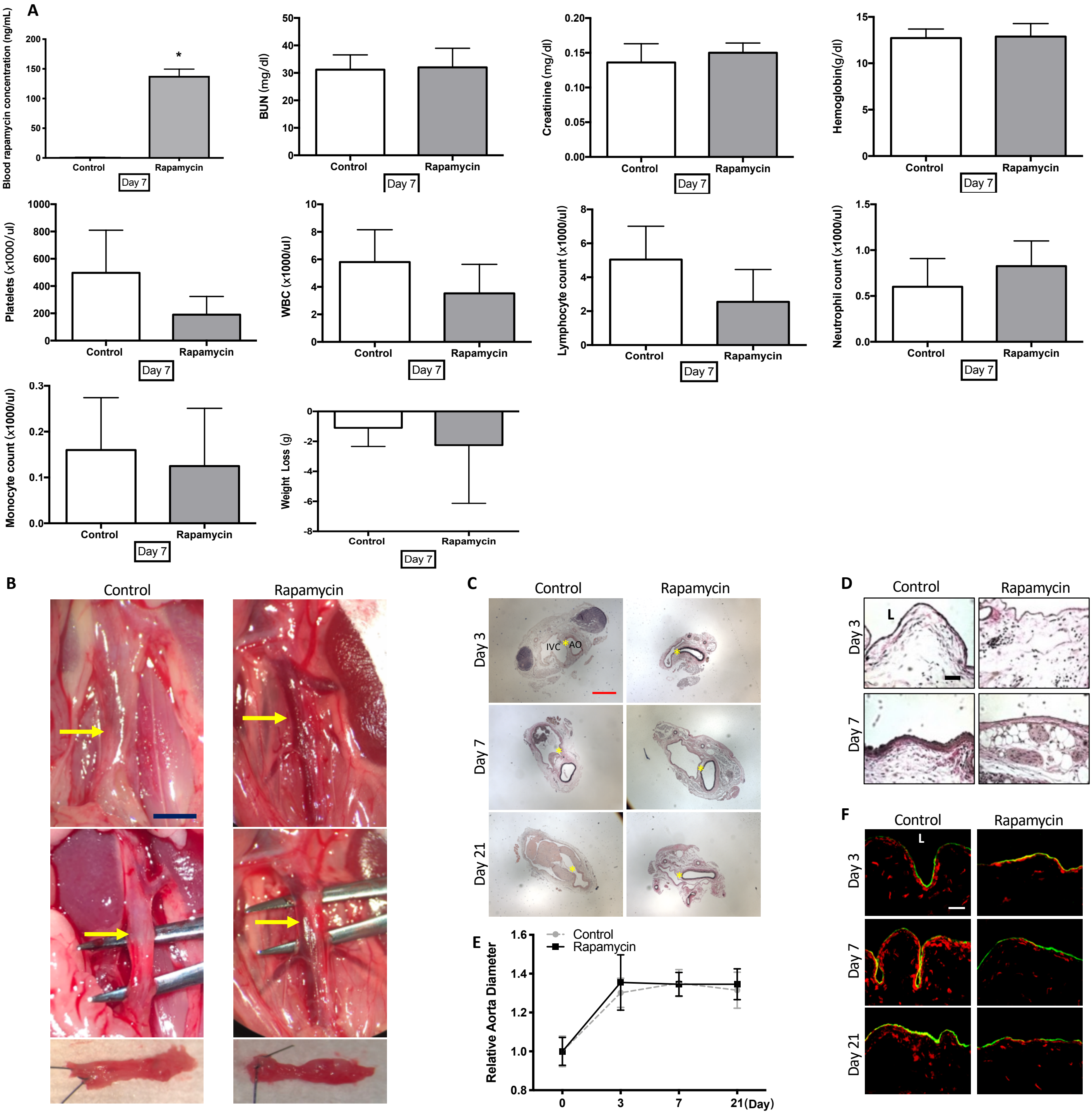

Supplementary Figure 1. Reduced AVF wall thickness, extracellular matrix deposition, SMC and macrophages with rapamycin. (A) Bar graphs showing quantification of serum rapamycin concentration, *P<0.0001 (t test); BUN, p=0.8506 (t test); creatinine, p=0.3830 (t test); hemoglobin, p=0.8502 (t test); platelet, p=0.1116 (t test); white blood cell, p=0.1763 (t test); lymphocyte, p=0.0977 (t test); neutrophil, p=0.2924 (t test); monocyte, p=0.6748 (t test); weight loss, p=0.5467 (t test) after control or rapamycin treatment in mice (day7); n=4-5. (**B**) Top panel: aortocaval fistula in mice treated with control vs rapamycin; middle panel: retroperitoneal tissue dissected to obtain proximal control of the aorta and IVC; bottom panel: extracted AVF tied just below the renal veins; arrow denotes IVC; scale bar, 1 cm. (**C**) Cross-section of AVF just below the renal veins in mice treated with control vs rapamycin; *: AVF; AO: aorta; scale bar, 100μm. (**D**) Representative photomicrographs showing AVF wall thickness in mice treated with control vs rapamycin. Scale bar, 25 μm. L, lumen. (**E**) Line graph showing relative AVF arterial diameter in mice treated with control or rapamycin; normalized to day 0; p=0.534 (ANOVA). n=5-6. (**F**) Representative photomicrographs showing dual immunofluorescence (IF) for ICAM-1 (green) and p-eNOS (red) in AVF after control or rapamycin treatment; day 3, day 7 and day 21.

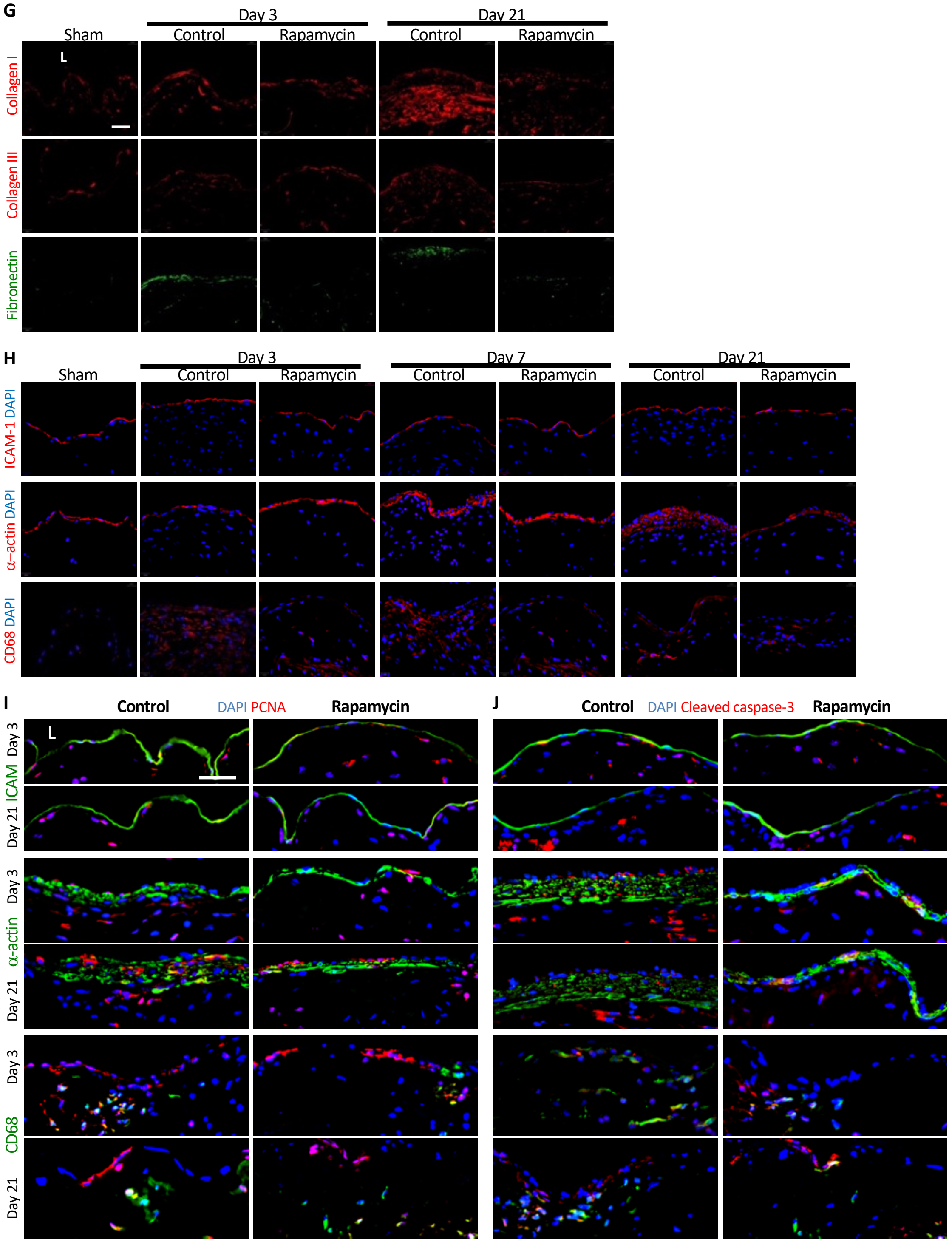

Supplementary Figure 1 (continued). (G) Representative IF photomicrographs showing extracellular matrix immunoreactivity in control or rapamycin treated groups. Collagen I and III (red) and fibronectin (green). (**H**) Representative IF photomicrographs of ICAM-1 (top row), α-actin (middle row) and CD68+ cells (bottom row) in control or rapamycin treated mice AVF. (**I**) Photomicrographs showing representative IF of PCNA (red) merged with ICAM, α-actin or CD68 (green), and DAPI (blue) in AVF of control vs rapamycin treated mice (day 3 and 7); L, lumen; scale bar, 25 μm. (**J**) Photomicrographs showing representative IF of cleaved caspase-3 (red) merged with ICAM, α-actin or CD68 (green), and DAPI (blue) in AVF of control or rapamycin treated mice (day 3 and 7); L, lumen; scale bar, 25 μm.

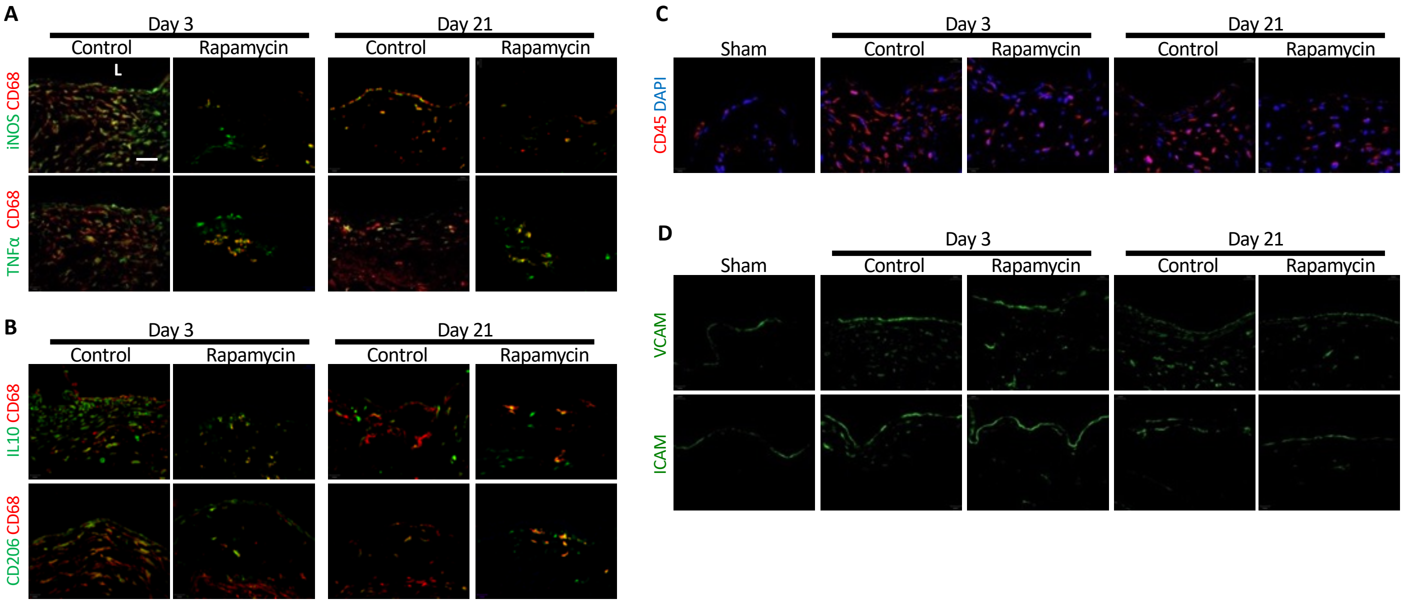

Supplementary Figure 2. Reduced M1- and M2-type macrophages with rapamycin. (**A**) Representative photomicrographs showing dual IF for CD68 (red) and iNOS (green, top row), TNF-α (green, bottom row). (**B**) IL10 (green, top row) or CD206 (green, bottom row) in AVF after control or rapamycin treatment. (**C**) Representative IF photomicrographs of CD45+ cells. Scale bar, 25 μm. L, lumen. (**D**) Representative IF photomicrographs of VCAM-1 (top row) and ICAM-1 (bottom row) in the AVF after control or rapamycin treatment.

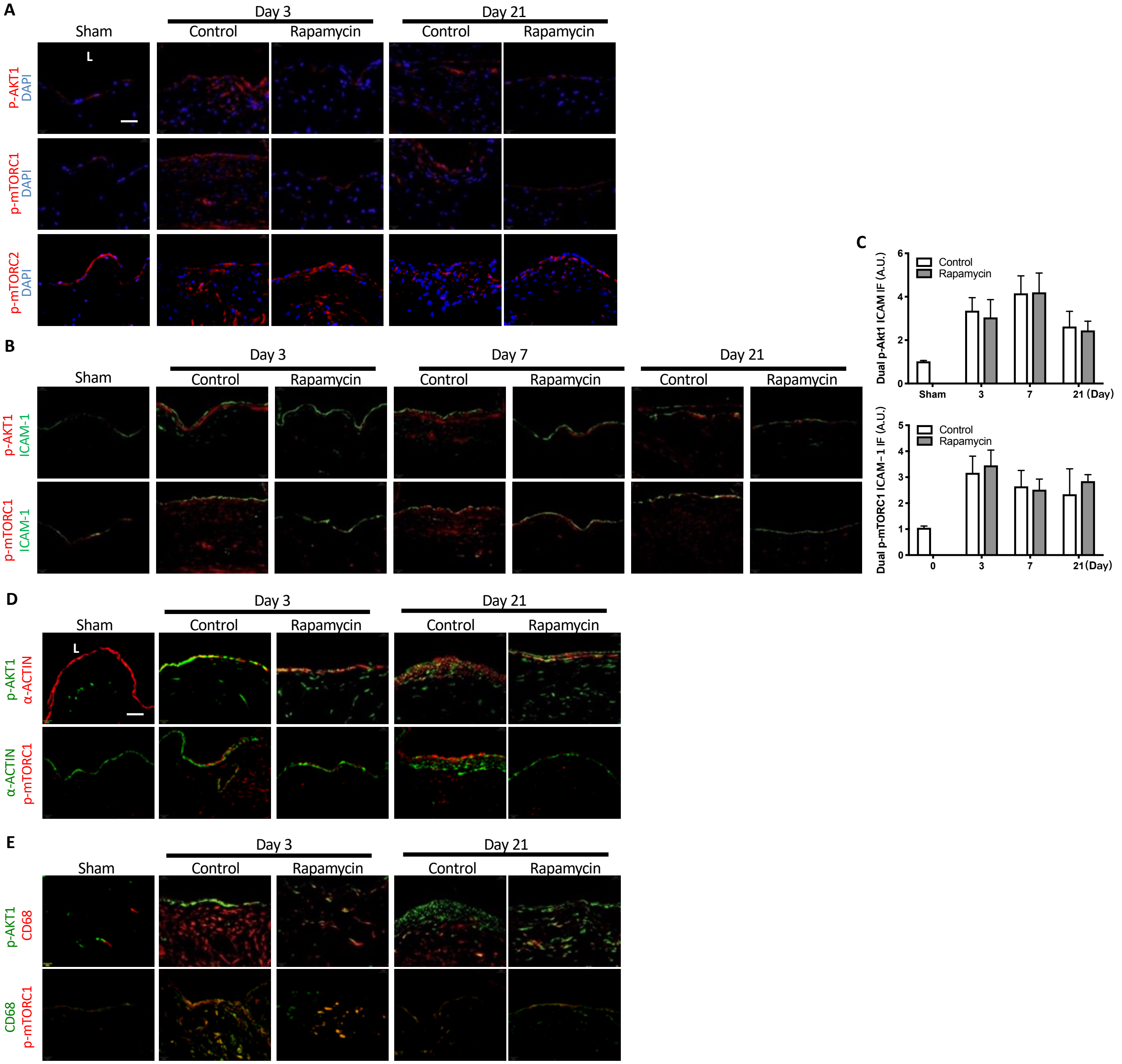

Supplementary Figure 3. Reduced Akt1 and mTORC1 but not mTORC2 phosphorylation with rapamycin. (**A**) Representative IF photomicrographs of p-Akt1+ (top), p-mTORC1+ (middle) and p-mTORC2+ (bottom) cells in control or rapamycin treated mice AVF; sham, day 3 and day 21. Scale bar, 25µm. L, lumen. (**B**) Representative photomicrographs showing dual IF for ICAM-1 (green) and p-Akt1 (red, first row) or p-mTORC1 (red, second row) in AVF after control or rapamycin treatment; sham, day 3, day 7 and day 21. (**C**) Bar graphs showing quantification of dual IF after control or rapamycin treatment. p-Akt1-α-actin: p=0.2036 (ANOVA); n=4-5. p-mTORC1-ICAM: p=0.4876 (ANOVA); n=4. (**D**) Representative photomicrographs showing dual IF for α-actin (red) and p-Akt1 (green, top row), α-actin (green) and p-mTORC1 (red, bottom row), (**E**) CD68 (red) and p-Akt1 (green, first row), CD68 (green) p-mTORC1 (red, second row) in AVF after control or rapamycin treatment; sham, day 3 and day 21.

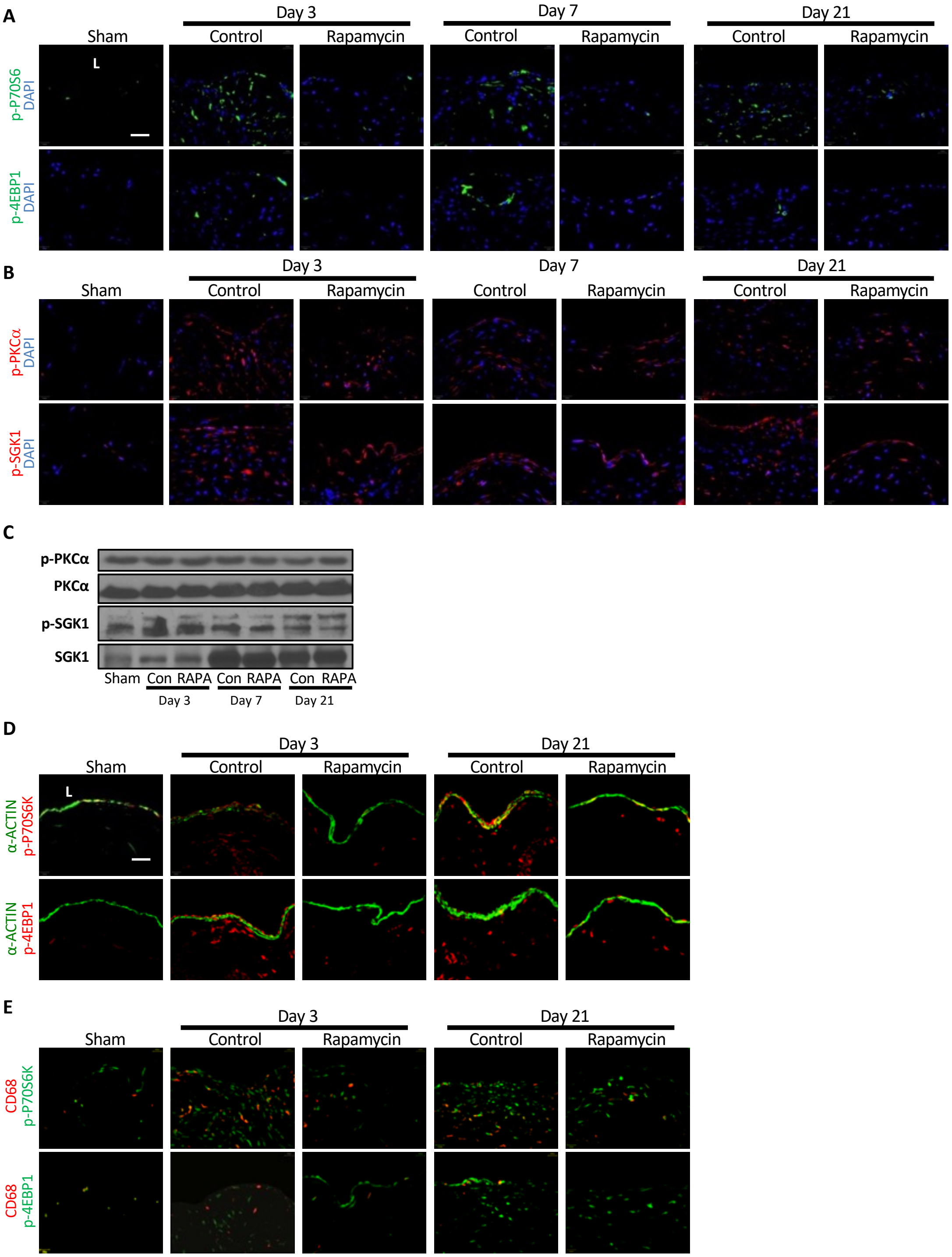

Supplementary Figure 4. Reduced p70S6K1 and 4EBP1, but not PKCα or SGK1, phosphorylation with rapamycin. (**A**) Photomicrographs of representative IF images of p-p70S6K1+ and p-4EBP1+ cells in control or rapamycin treated mice AVF. Scale bar, 25μm. L, lumen. (**B**) Photomicrographs of representative IF images of p-PKCα+ and p-SGK1+ cells in control or rapamycin treated mice AVF. Scale bar, 25μm. L, lumen. (**C**) Representative Western blot showing phosphorylation level of PKCα and SGK1 after control or rapamycin treatment. (**D**) Photomicrographs of representative IF images for α-actin (green) and p-P70S6K1 (red, top row) or p-4EBP1 (red, bottom row) as well as (**E**) CD68 (red) and p-P70S6K1 (green, top row) or p-4EBP1 (green, bottom row) in AVF after control or rapamycin treatment.

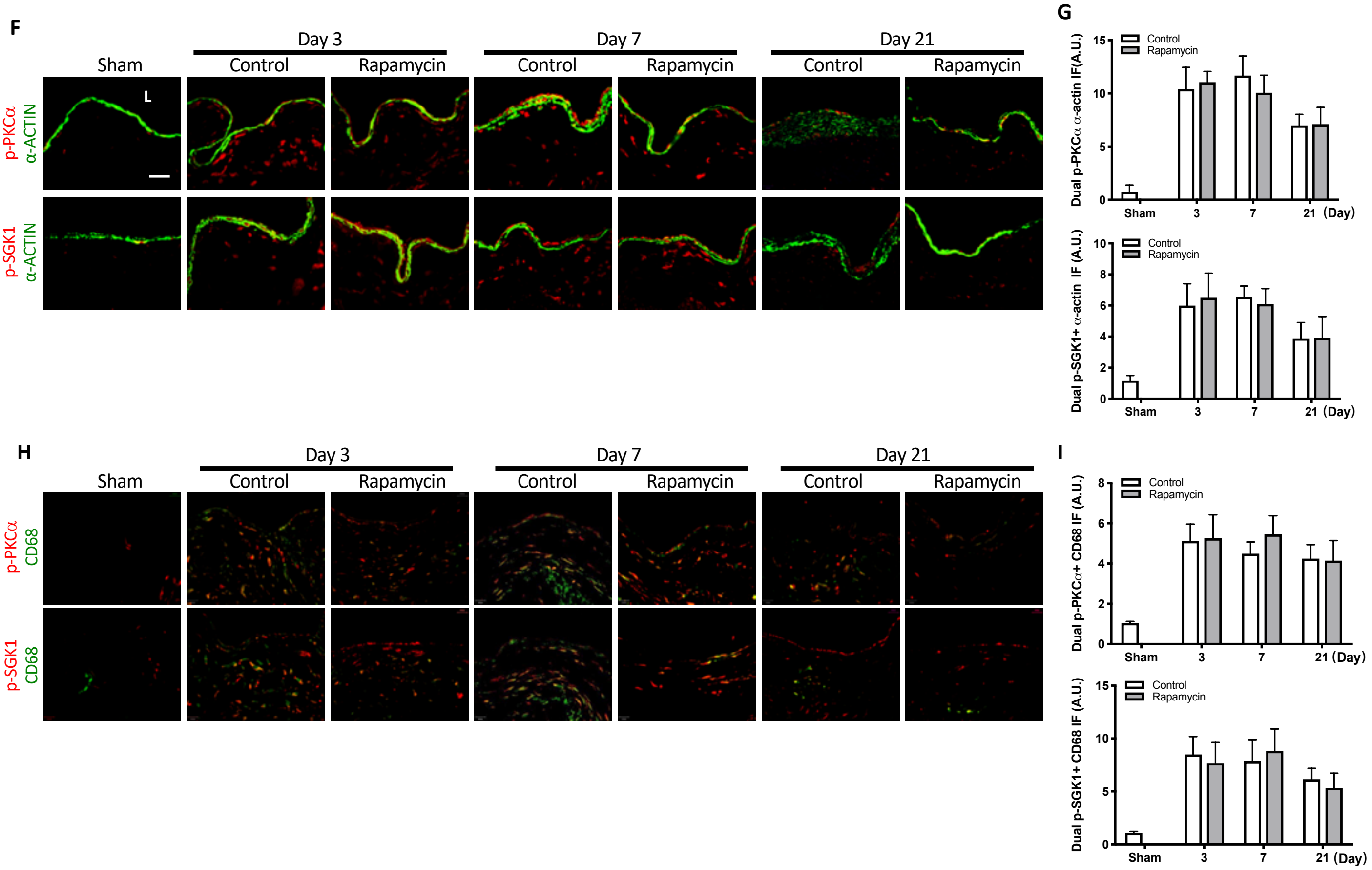

Supplementary Figure 4 (continued). Reduced p70S6K1 and 4EBP1, but not PKCα or SGK1, phosphorylation with rapamycin. (**F**) Microphotographs of representative IF images for α-actin (green) and p-PKCα (red, top row) or p-SGK1 (red, bottom row) in AVF after control or rapamycin treatment. (**G**) Bar graphs showing quantification of dual IF after control or rapamycin treatment, normalized to sham. p-PKCα-α-actin: p=0.6597 (ANOVA); n=4-5. p-SGK1-α-actin, p=0.01024 (ANOVA); n=4-5. (**H**) Photomicrographs of representative IF images for CD68 (green) and p-PKCα (red, top row) or p-SGK1 (red, bottom row) in AVF after control or rapamycin treatment. (**I**) Bar graphs showing quantification of dual IF after control or rapamycin treatment, normalized to sham. p-PKCα-CD68: p=0.3697 (ANOVA). p-SGK1-CD68, p=0.3341 (ANOVA. n=4-5.

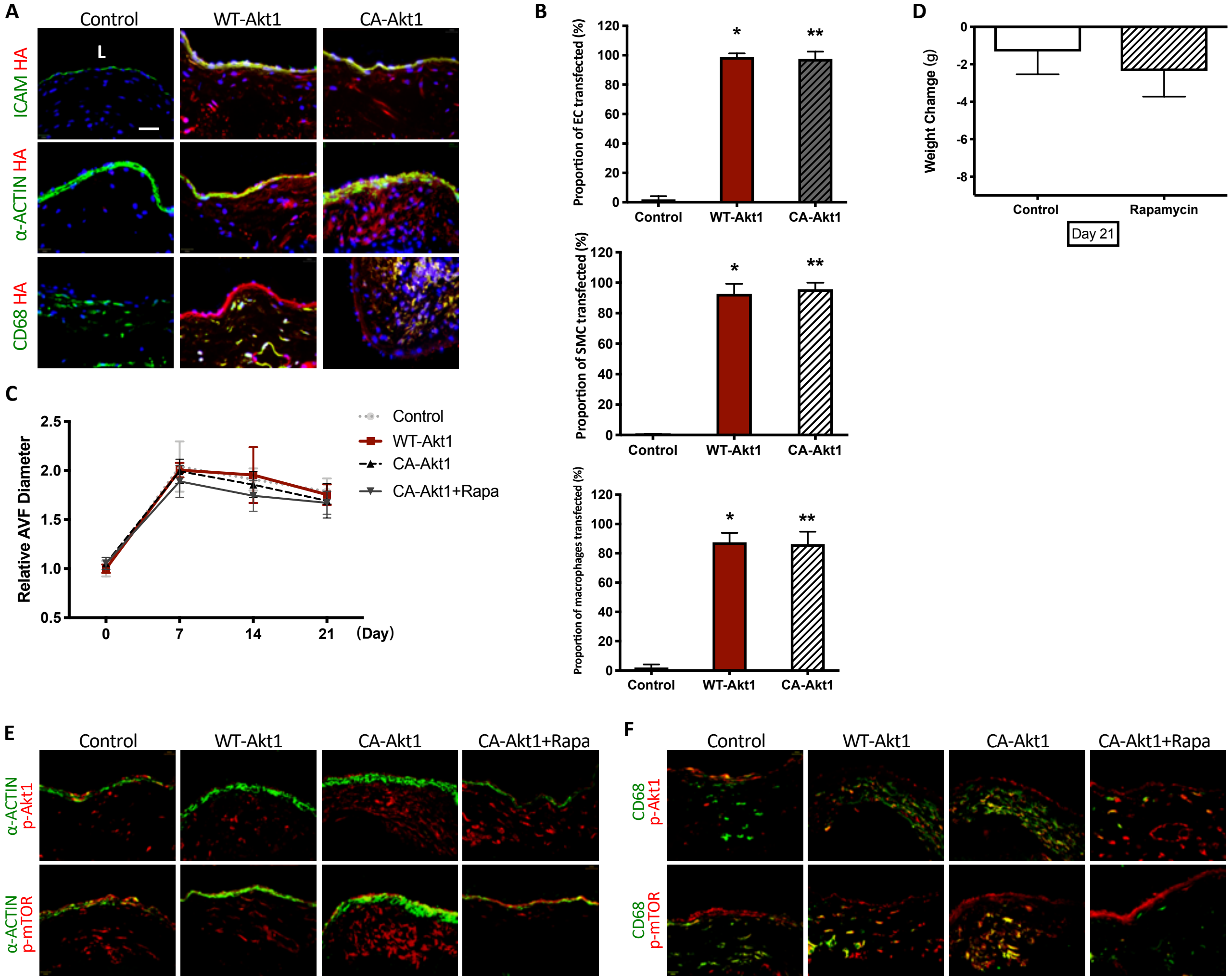

Supplementary Figure 5. Rapamycin inhibits mTORC1 phosphorylation during venous remodeling. (**A**) Representative photomicrographs showing dual IF for α-actin, CD68 or ICAM (green) and HA (red) in AVF after adventitial delivery of control, WT-Akt1, and CA-Akt1; day 21. Scale bar, 25μm. L, lumen. (**B**) Bar graphs showing proportion of HA-ICAM+ to total ICAM+, HA-α-actin+ to total α-actin+, and HA-CD68+ to total CD68+ cells after adventitial delivery of control, WT-Akt1, and CA-Akt1; normalized to control. HA-ICAM: p=0.0012 (ANOVA); Control vs. WT-Akt1: *, p=0.0136; Control vs. CA-Akt1: **, p=0.0064 (post hoc); n=4. HA-α-actin: p=0.0030 (ANOVA); Control vs. WT-Akt1: *p<0.0001; Control vs. CA-Akt1: **p<0.0001 (post hoc); n=4. HA-CD68: p=0.0010 (ANOVA); Control vs. WT-Akt1: *p=0.0006, Control vs. CA-Akt1: **p=0.0004 (post hoc); n=4. (**D**) Bar graphs showing quantification of weight loss, p=0.1926 (t test) after control or rapamycin treatment in mice (day 21); n=4-5. (**C**) Line graph showing AVF diameter in mice treated with control, WT-Akt1-Ad, CA-Akt1-Ad or CA-Akt1-Ad with rapamycin. p=0.1817 (ANOVA). n=4–5. (**E**) Representative photomicrographs showing dual IF for α-actin (green) and p-Akt1 (red, top row) or p-mTORC1 (red, bottom row) as well as (**F**) CD68 (green) and p-Akt1 (red, top row) or p-mTORC1 (red, bottom row) in AVF after local delivery of control, WT-Akt1, CA-Akt1, and CA-Akt1 with daily 250 μg IP rapamycin injection (day 21).

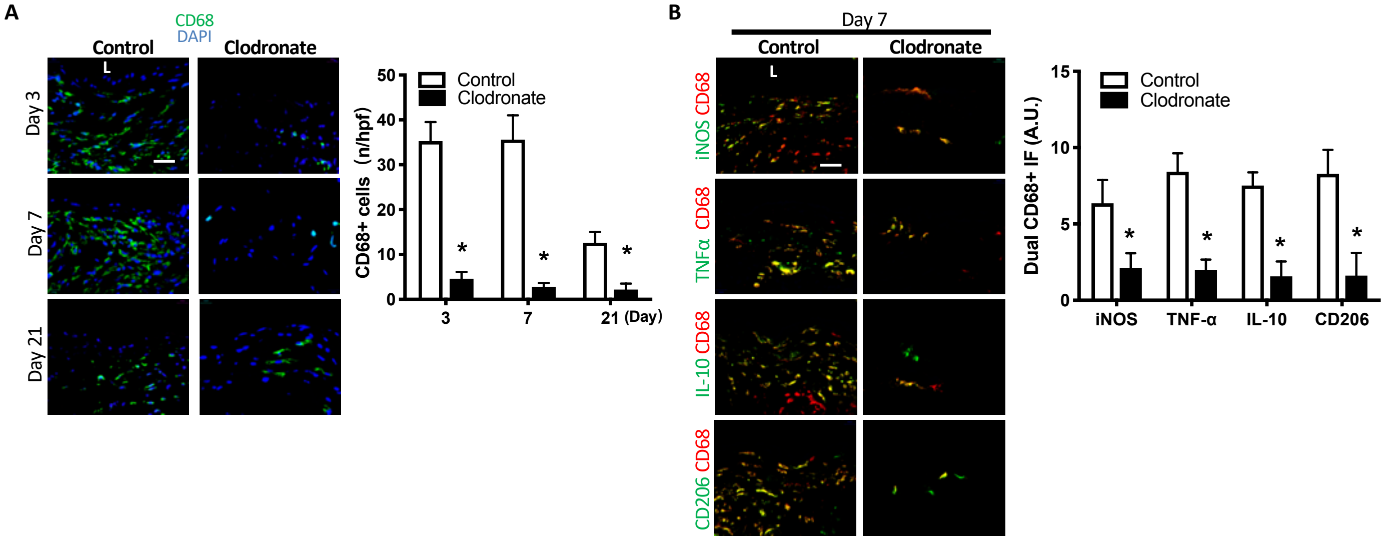

Supplementary Figure 6. Macrophage depletion is associated with reduced AVF wall thickness and patency. (**A**) Representative photomicrographs showing CD68+ immunoreactive cells in mice treated with control or clodronate. Scale bar, 25µm. L, lumen. Bar graphs showing number of CD68+ cells in AVF after control or clodronate treatment. *p<0.0001 (t test). n=5. (**B**) Representative photomicrographs showing dual IF for CD68 (red) and iNOS (green, top row), TNF-α (green, second row), IL-10 (green, third row) or CD206 (green, bottom row) in AVF after control or rapamycin treatment; day 7. Scale bar, 25 µm. L, lumen. Bar graphs showing quantification of dual IF after control or clodronate treatment (day 7). CD68-iNOS: p=0.01351 (t test). CD68-TNF-α: p<0.0001 (t test). CD68-IL-10: p<0.0008 (t test). CD68-CD206: p=0.0011 (t test). n=5.

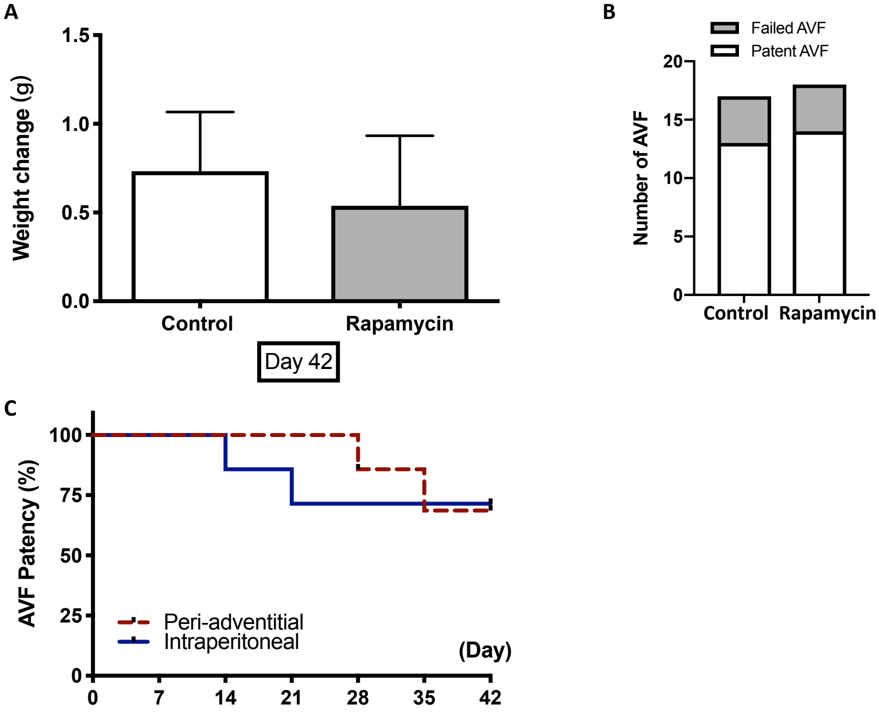

Supplementary Figure 7. Rapamycin treatment is associated with reduced AVF wall thickness but increased AVF patency. (**A**) Bar graphs showing quantification of weight change, p=0.1977 (t test) after control or rapamycin treatment in mice (day 42); n=12-13. (**B**) Technical success rate of AVF creation in rapamycin treated group (77.8%; 14/18) compared to control (76.5%; 13/17); P=0.9871 (chi-square). (**C**) Line graph showing AVF patency rate in mice treated with IP rapamycin or adventitial delivery of a single dose of rapamycin via pluronic gel. *P = 0.9027 (Log-rank), n=6-7 in each arm.

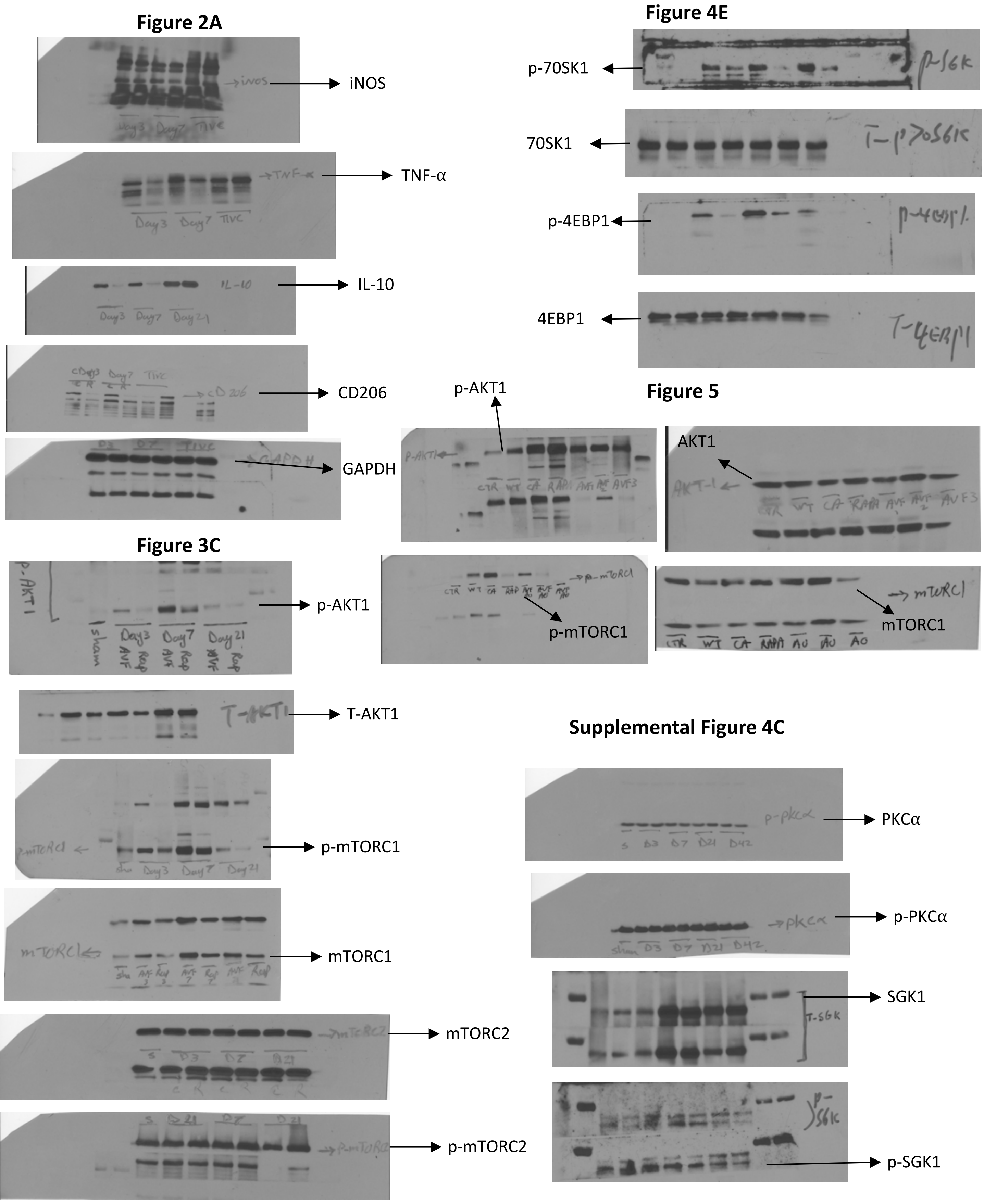

Supplementary Figure 8. Unedited Gels shown in order of their presentation in the manuscript
